# Home security cameras as a tool for behavior observations and science affordability

**DOI:** 10.1371/journal.pone.0360298

**Published:** 2026-10-26

**Authors:** Billie C. Goolsby, Marie-Therese Fischer, Tony G. Chen, Daniela Pareja-Mejía, Daniel A. Shaykevich, Amaris R. Lewis, Gaelle Raboisson, Katharina Dellefont, Madison P. Lacey, Lauren A. O'Connell

**Affiliations:** 1 Department of Biology, Stanford University, Stanford, California, United States of America; 2 Department of Mechanical Engineering, Stanford University, Stanford, California, United States of America; 3 Graduate Program in Zoology, Universidade Estadual de Santa Cruz, Bahía, Brazil; Oklahoma State University, UNITED STATES OF AMERICA

## Abstract

Reliably capturing transient animal behavior in the field and laboratory remains a logistical and financial challenge, especially for small ectotherms. Here, we evaluate home security cameras as affordable, accessible and suitable alternatives for monitoring small, cold-blooded animals historically overlooked by commercial camera traps. These cameras are often lightweight, weather-resistant, operate offline or online, store data continuously for up to four weeks and can use phone notifications over local Wi-Fi to alert users to behaviorally relevant events. We first benchmark a representative home security camera against an established trail camera under matched laboratory conditions in both continuous and motion-triggered modes, comparing event detection, recording duration, and missed-event rates. We then validate the system during a 1.5-month deployment in a tropical primary rainforest, assessing weather durability, sensitivity-versus-data-volume trade-offs, and whether behavioral measurements obtained in the laboratory are reproducible under natural conditions. We discuss the relative affordability of our system for researchers in the Global South, home to the largest ectotherm diversity.

## Introduction

Accurately quantifying animal behavior is critical for understanding how organisms respond to challenges and opportunities in their environment, which influences the fitness and survival of a species. Human presence can obscure natural behaviors [1, 2], and remote cameras bypass the need for physical presence of the researcher and allows for operationalization of behaviors [3]. Remote camera approaches are used to study behavior at both population [4, 5] and individual levels [6, 7]. Mammals and birds are the most common fauna monitored with cameras in the wild, but only a small range of prior studies (<2%) have focused on ectotherms, specifically amphibians and reptiles [8–10]. This gap between ectotherms and endotherms can be explained by both technical and logistical difficulties: many camera traps rely on surface body heat signatures (passive infrared, PIR) warmer than the surrounding environment [11]. Ectotherms do not produce endogenous body heat higher than the surrounding environment, making PIR camera traps unreliable without experimental background modifications for maximizing heat contrast [10, 12, 13], which is difficult in remote areas or when minimal environmental interference is desired. Moreover, countries in the Global South hold much of the world’s ectotherm biodiversity [14], yet accessibility and affordability of camera traps for local researchers are prohibitive for this research tool [15]. Indeed, tropical biomes, which are at highest defaunation risk, are disproportionately less monitored with camera traps compared to other environments [15]. Increasing camera weather resistance correlates with higher prices, and many camera traps rely on cellular networks headquartered in the Global North, which can present difficulties in network-limited landscapes [16]. An ideal camera system should be affordable, capable of detecting small ectotherm movement, weather-resistant, and operational in the absence of electricity and phone connectivity.

Home security cameras, originally designed for the residential market, offer an affordable alternative class of recording device that can be repurposed for research. These cameras frequently use pixel change detection, which compares pixel values between successive video frames, rather than infrared detection, to trigger recordings [17]. In daylight, home security cameras use natural lighting for pixel comparisons, and at night, use an active IR system converted into a greyscale image used for pixel change. This enables detection of ectotherm movement, whose body temperature does not differ markedly from ambient conditions. Several home security models are weather-resistant, operate offline, and connect to local Wi-Fi networks rather than depending on cellular providers, which is a key advantage in tropical field sites where Global North cellular coverage is poor or absent.

Establishing whether such cameras are suitable for ectotherm behavioral research requires two complementary lines of evidence. First, the affordable camera must perform comparably to established commercial systems under controlled conditions, where independently verified behavior is available for direct comparison. Second, the camera must remain functional and produce ecologically valid data under the real-world conditions, including humidity and temperature extremes, offline operation, and free-ranging animals in complex natural backgrounds.

Here, we provide a technical comparison of seven camera systems currently available on the United States market, including affordable home security cameras and camera traps commonly used for research. We then present a two-stage evaluation of a representative system (Wyze v3) for ectotherm behavioral research. First, we benchmark the security camera against an established commercial trail camera (GardePro A3S) in a laboratory terrarium to determine (i) how the two systems compare in the number of behavioral events detected, (ii) how the cameras compare in the duration of behavior recorded per event, and (iii) what fraction of events are recorded by one camera and missed by the other (Study 1). We next validated the system in the field during a 1.5-month deployment in a tropical primary rainforest in a rubric with three independent criteria: hardware survival under tropical conditions, the trade-off between motion-detection sensitivity and event capture, and the ecological validity of laboratory-measured behavioral metrics by comparing paternal visit duration in poison frogs between captive and free-ranging individuals (Study 2).

We used two poison frog species as study systems to address different aspects of camera-assisted behavioral monitoring: the relatively large dyeing poison frog (*Dendrobates tinctorius*) served as our laboratory benchmarking system because its larger vivaria could accommodate both camera systems simultaneously, allowing direct comparison while monitoring adult behavior. The variable poison frog (*Ranitomeya variabilis*) provided a field-relevant system for monitoring parental care and tadpole development. Males transport newly hatched tadpoles to small water-filled leaf axils, where the cannibalistic tadpoles develop individually. These discrete, abundant, and accessible aquatic nurseries provide natural observational units that can be readily monitored and manipulated, making *R. variabilis* particularly suitable for evaluating camera-based monitoring of parental care and tadpole behavior under field conditions.

We hypothesized that the Wyze v3 would outperform the GardePro A3S in detecting and recording ectotherm behavioral events because its pixel-change detection is better suited to small ectotherms than passive infrared triggering. However, given its substantially lower cost and design as a consumer home security camera rather than a research-grade camera trap, we expected this improved behavioral performance to come at the cost of reduced hardware reliability under tropical field conditions. We further predicted that behavioral measurements obtained with pixel-change detection in captivity would be comparable to those obtained from free-ranging animals.

## Materials and methods

### Study 1: Camera comparison and laboratory benchmarking

#### Camera systems compared.

We compiled technical specifications and availability of seven camera systems (Table 1) to evaluate their suitability for lab and field studies of amphibian behavior: three commercial trail or monitoring cameras (Reconyx Hyperfire 2 [Holmen, WI, USA]; Bushnell Nature View HD [Overland Park, KS, USA], used in [10]; GardePro A3S [Hong Kong], used in [18]), three home security cameras (Wyze v3 [Seattle, WA, USA], Google Nest [Mountain View, CA, USA], used in [19, 20]; Tapo C120 [Irvine, CA, USA]), and a manual handheld camera (Sony HDR-CX405 [Tokyo, Japan], used in [21, 22] respectively. To select two cameras for direct benchmarking, our criteria were ectotherm detectability (pixel-change vs. passive infrared sensors (PIR)), affordability, and local-network availability. We opted to compare the Wyze v3 and GardePro A3S camera systems, as they were the most affordable representatives of their respective classes at the time of the study, while sharing comparable motion-detection length, storage capabilities, and weather resistance.

**Table 1:** Camera performance for studying ectotherm behavior.

System Specifications	Consequence on Amphibian Studies	Wyze Camera v3*	Google Nest Camera, Indoor & Wired	Tapo C120	Sony Camera HDR-CX405	Reconyx Hyperfire 2 Cellular Professional Covert IR Camera	Bushnell Nature View HD	GardePro A3S Trail Camera
Ability to notify phone with local network	Quickly alerts researchers to behavior	Yes	Yes	Yes	No	Yes	No	No
Ability to notify phone without local network	Quickly alerts researchers to behavior	No	No	No	No	No	No	No
Cellular Source	Alerts rely on either local networks or cellular ones	2.4 GHz local Wifi	2.4GHz/5GHz Wi-Fi	IEEE 802.11b/g/n, 2.4 GHz Wi-Fi	None	3-4G LTE (ATT & Verizon, some international plans)	None	None
Price (USD)	Cost influences number of cameras purchasable	$39.98	$99.99	$35.99	$229.99	$459.99	$299.99	$89.99
Image Resolution	Equivalent across most cameras	HD 1920 x 1080p	HD (1920 x 1080)	2K QHD 4MP (2560 × 1440 px)	2.1 Megapixels (1920 x 1080)	1080P HD Widescreen	HD 1080P	48 Megapixels HD
Video Quality and sound	Equivalent across most cameras	HD 1920 x 1080p	HD (1920 x 1080)	2K QHD 4MP (2560 × 1440 px, 20 fps)	1920 x 1080/60p	720P HD Video with Audio (cellular enabled cameras do not transmit video)	HD 1920x1080p	2304 x 1296P@20fps (16:9 OR 1920 x 1080P@30fps (16:9) OR 1280 x 720P@30fps (16:9)
Detection Method (Day)	Risk of not detecting amphibians with PIR	Pixel change	Pixel change	1/2.9” Progressive Scan CMOS Starlight Sensor	Exmor R CMOS Sensor	PIR	PIR	PIR (high, normal low)
Motion Detection Video length	Variable, depends on research goal	Up to 5 minutes per notification	Up to 3 hours of event history totally	Up to 3 minutes per notification	None, manual	Up to 90 seconds per notification	up to 60s	Up to 5 minutes
Continuous Video length	Remote monitoring of behaviors that may last longer than several minutes.	24/7 for up to 4 weeks with SD card	24/7 for up to 10 days with subscription	24/7 for up to 4 weeks with SD card	Approximately 2 hrs 35 minutes of battery life	No continuous recording abilities (trigger only)	No continuous recording abilities (trigger only)	No continuous recording abilities (trigger only)
Detection Method (Night and wavelength in nm)	IR wavelength that can detect amphibians	Near-IR (940) and Far-IR (850)	Far-IR (850)	Near-IR (940) and Far-IR (850)	None explicitly stated	No-Glow™ High Output Covert IR	PIR	PIR (940nm) No Glow IR LEDs
Camera weight (g)	Lower weights are more carryable for fieldwork	98.8	393	100	190-215	~750	246.6	530
IP Score	IP66 best for heavy pressurized water exposure, IP65 handles rain	IP65	None provided.	IP66	None provided.	IP66	IP66	IP66
Operating Temperatures (°C)	Most cameras tolerate most biome temperature ranges	−20 - + 40	0- + 40	−25 - + 45	None provided.	−40 - + 60	−4 - + 140	−20 - + 60
Night vision distance (m)	Depends on animal of interest	None provided	15	30	None provided.	45	18.3	30
Cloud saving abilities	Depends on researcher needs	Yes, with extra fees	Yes, with extra fees	Yes, with extra fees	No	No	No	No
Highest amount of internal memory allowed on camera via SD card (Gb)	Higher Gb allows for cameras being left alone longer	512	Up to 1 hr of internal storage (no Gb amount provided).	512	No limit for SD card	512	32	512
Power source (years last)	USB requires continuous power source, which needs to be resolved before field work	USB	USB	USB	Battery or USB	12 AA (2 yrs)	AA (4–12) Up to one year	AA (8) Over a year depending on usage
Field of View	Depends on camera’s ability to orient and point towards area of interest	130°	135°	120°(Diagonal), 103°(Horizontal), 55°(Vertical)	90-270°	None provided	50°	Total 120° Central zone: 60°, Left side: 30°,Right side: 30°

We compare features and accessibility of three home security systems (Wyze, Google Nest, Tapo) against four traditional camera traps for animal behavior currently on the United States commercial market (Sony, Reconyx Hyperfire 2, Bushnell Nature View HD, GardePro). If cameras are no longer commercially available by the original vendor, we provide the most similar commercially available camera from the same vendor. We discuss each feature in the context of studies on amphibian behavior. Abbreviations: LTE: long term evolution (a standard for wireless data transmission), HD: high definition, PIR: passive infrared sensor, IR: infrared, CMOS: Active pixel sensor; IP65 and IP66: indices for ingress protection (weather resistance, the higher the better), AA: single cell dry battery. Dark grey indicates home security cameras. Prices retrieved March 2026.

* discontinued and replaced with Wyze v4 as of March 2026.

#### Camera setup and modifications in a laboratory setting.

Detailed setup instructions are available on GitHub (https://github.com/laurenoconnelllab/WyzeSetUps) and a modification protocol for close-range lens refocus on protocols.io (dx.doi.org/10.17504/protocols.io.dm6gp3p38vzp/v1). In brief, Wyze v3 cameras were attached by velcro to the side of Exoterra tanks in the laboratory, around 15.5 to 17.5 cm above the focal area, powered via USB through a Nexwell charging station, and recorded to 128 GB secure digital (SD) cards (filled in approximately 10 days, then swapped) (Fig 2A).

#### Anuran study system in the laboratory.

**Dyeing poison frog (*Dendrobates tinctorius*)**. Laboratory benchmarking was performed with *D. tinctorius* as their vivaria were large enough to fit both camera types simultaneously (Fig 1). One captive-bred adult male and one adult female were housed together in a 45.72 × 45.72 × 45.72 cm Exoterra terrarium with sphagnum moss, driftwood, live Pothos plants, and water-filled petri dishes (R/O Rx, Josh’s Frogs). Frogs were fed *Drosophila hydeii* flies dusted with vitamin mix and *Folsomia candida* springtails three times per week. Both cameras simultaneously filmed a communal water pool and Coco-hut shelter. The observation housing was set on a 12:12 light cycle, the average temperature and humidity of the observation was recorded for each day of observation, usually around 23.5°C and 95% humidity within the tank. Recording comparisons between camera systems occurred in the same tank to standardize light, temperature, and humidity.

**Fig 1:**
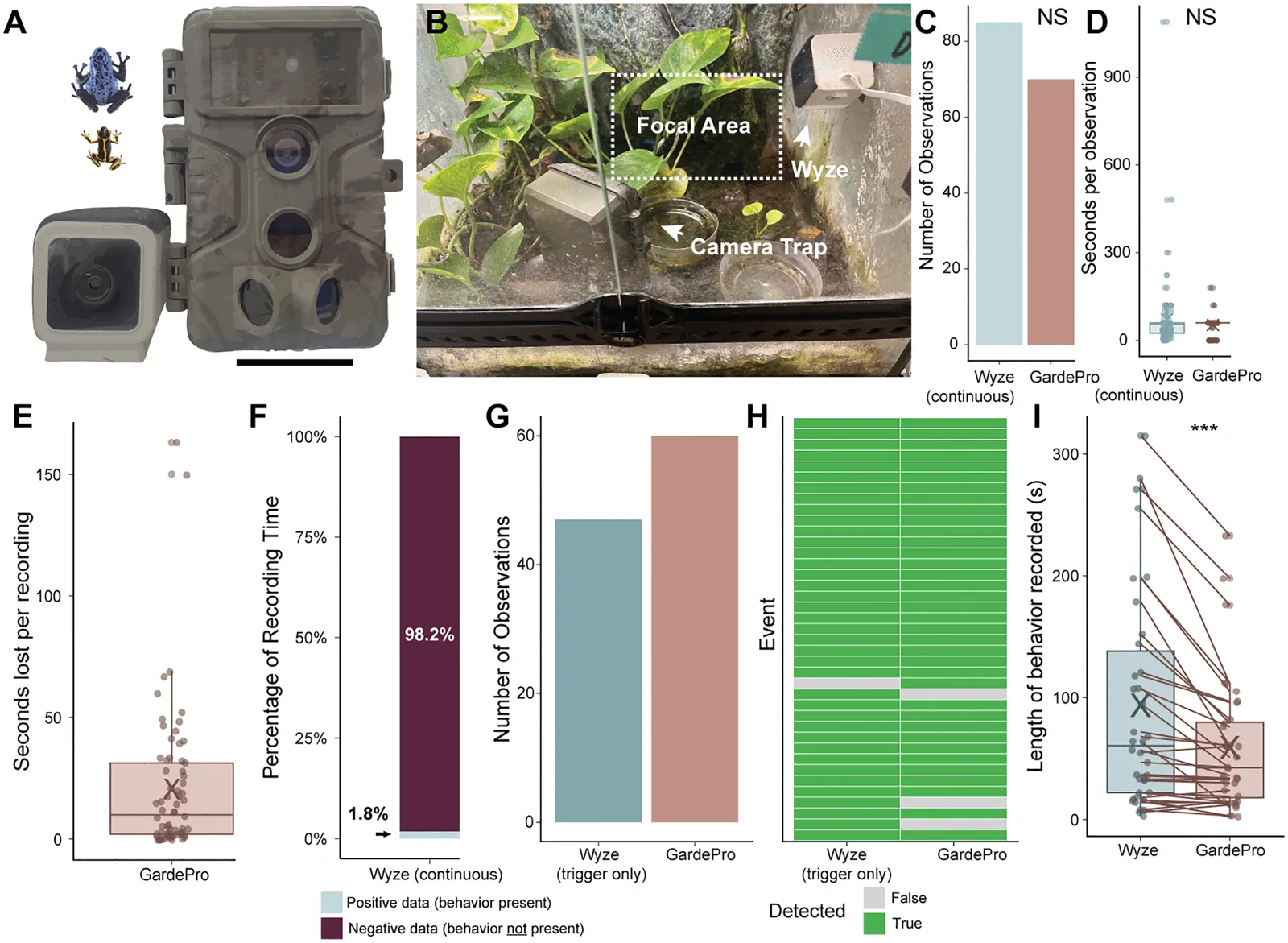
Comparisons of a home security camera (Wyze) in both continuous (24/7) and trigger formats versus a typical camera trap (GardePro) when recording the same area. **(A)** Size comparison of GardePro and Wyze v3 to two species of poison frogs, *Ranitomeya imitator* (bottom) and *Dendrobates tinctorius* (top). Scale bar coincides to ~52 mm. **(B)** We recorded *D. tinctorius* around a communal water pool and Coco-hut for a fairer comparison between both camera systems. **(C)** Number of observations detected by Wyze in continuous mode and GardePro (no significant difference, ns). **(D)** Observation length in seconds per recording detected by Wyze in continuous mode and GardePro (no significant difference, ns). **(E)** Due to Wyze recording in continuous mode, no data can be lost due to errors in triggering. However, 24/7 collection of data can produce an abundance of false negative data that takes a long time to parse through. On average, GardePro loses around 20 seconds of animal behavior per trigger compared to 24/7 recordings. We then compared this to Wyze’s continuous mode, which is constantly saving data. **(F)** In continuous mode, Wyze collects a disproportionately large amount of false positive data where no behavior is actually recorded (333900 seconds) compared to actual behavior (6153 seconds). **(G)** Wyze was triggered less often than GardePro, however, **(H)** GardePro fails to detect the same event that Wyze records slightly more often than Wyze fails to detect the same event that GardePro records. **(I)** Of the same events that GardePro and Wyze both successfully detected, Wyze recorded a significantly longer interval of behavior (*p < 0.001*, V = 631, Wilcoxon signed rank test with continuity correction). To ensure equity in camera comparisons, we summed the amount of time GardePro or Wyze recorded total behavior per event, rather than per individual file, which would be due to storing mechanisms specific to each camera.

#### Laboratory benchmarking.

We benchmarked the Wyze v3 against the GardePro A3S in a laboratory terrarium housing a captive-bred adult male and adult female Dyeing poison frog (*Dendrobates tinctorius*). Both cameras simultaneously filmed the same focal area: a communal water pool and shelter. We ran two separate 96-h benchmarking periods: (i) Wyze in continuous (24/7) mode vs. GardePro in trigger-only mode (GardePro has no continuous option), and (ii) Wyze in trigger-only mode (75% motion sensitivity) vs. GardePro in trigger-only mode. For the continuous comparison, we quantified the number of behavioral observations per camera, the duration of behavior per observation, and the seconds of behavior lost per recording by GardePro relative to the continuously recording Wyze. We also measured the proportion of total Wyze recording time that contained no behavior. For the trigger-against-trigger comparison, we quantified the number of triggers per camera, the event-by-event concordance of detection (i.e., for each behavioral event, whether each camera detected it), and the duration of behavior recorded per event for events successfully detected by both cameras. To equitably compare cameras, we summed total recorded behavior per event, rather than per individual file, to control for camera-specific file-storage mechanisms.

#### Study 2: Field validation.

Laboratory benchmarking establishes performance under controlled conditions but does not establish whether a camera survives field deployment, whether the settings required to keep datasets manageable cause unacceptable event loss, or whether laboratory-derived behavioral measurements remain valid for free-ranging animals. The primary rainforest site is characterized by Clusia tree patches on bare granite rock with daily rainfall fluctuating between 0 and 30 mm and temperatures of 20°C to 49°C, measured at exposed rock surfaces in direct sunlight [23] (Fig. S3 in S1 File). The site is inhabited by a population of the variable poison frog *Ranitomeya variabilis* (CITES Appendix II, IUCN Least Concern), in which males defend territories and shuttle hatched tadpoles to bromeliad leaf-axil pools where the tadpoles develop. Male *R. variabilis* exhibit uniparental care (clutch attendance, tadpole transport, nursery surveillance) [24, 25]*.* This species was selected for field validation because of its small size (~18 mm) and because its parental behavior involves transient, hard-to-observe events occurring at small spatial scales, posing a technically and ecologically relevant challenge for camera systems.

Validation was structured around three independent criteria: (1) Hardware survival: we deployed 20 Wyze v3 units for 1.5 months and recorded the number of unit failures and their causes; (2) Sensitivity-versus-event-capture trade-off: we positioned ten cameras above bromeliad leaf-axils after temporarily removing inhabiting tadpoles to free preferred deposition sites, and independently verified deposition events by scoring the daily presence or absence of newly deposited tadpoles, then compared the count of independently verified events to the count captured at lowest motion sensitivity; (3) Ecological validity of laboratory-measured behavior: we compared male parental visit durations to tadpole nurseries between captive (laboratory colony) and wild (Inselberg) *R. variabilis*. Captive observations were obtained from two breeding pairs in 45.72 × 30.48 × 30.48 cm Exoterra (Rolf Hagen, Baie D’Urfé QC, Canada) terraria. Wild observations were obtained from monitoring five tadpoles in bromeliad leaf-axil pools for 10 days. Approval was granted by the scientific committee of the Nouragues Ecological Research Station and Stanford University IACUC (protocol #33691); captive recordings were approved under protocols #34242 and #34304.

#### Anuran study system in the field.

**Variable poison frog (*****Ranitomeya variabilis***). The comparison of laboratory and field studies was performed using *R. variabilis*. Adult males defend territories and shuttle hatched tadpoles to bromeliad pools where the cannibalistic tadpoles develop over around three months [24–26]. The species is CITES Appendix II, IUCN Least Concern. Captive frogs were housed in Exoterra terraria with film canisters as egg-deposition sites and water-filled canisters as tadpole nurseries. Wild frogs were monitored on the Inselberg of the Nouragues Nature Reserve, French Guiana (4°5’ N, 52°41’ W), a primary rainforest site.

#### Camera setup and testing of hardware survival under tropical conditions.

In the field, cameras were configured at the field station with Wi-Fi (motion-triggered mode, lowest sensitivity, sound detection and notifications off), then deployed offline. Cameras were secured to bromeliad leaves 20–40 cm above the water reservoir housing a tadpole using rubber bands. Units deployed on exposed rock surfaces were additionally shielded from rain with simple plastic strips fixed above the openings without covering them to prevent moisture ingress while not preventing rain from entering the tadpole nurseries. Cameras were powered by 26,800 mAh power banks wrapped in absorbent diapers sheltered by two layers of inexpensive plastic zip-lock bags with openings facing into opposite directions; this low-cost solution proved sufficient over the deployment period and could be replaced by outdoor waterproof boxes if needed (Fig 2B). SD cards and power banks were replaced every two days. A phone displaying the accurate time at startup was filmed to correct for the timestamp reset that occurred at each power cycle. Cameras recorded freely behaving animals in their home enclosures and in natural habitats and no animals were sacrificed or anesthetized in this study.

**Fig 2:**
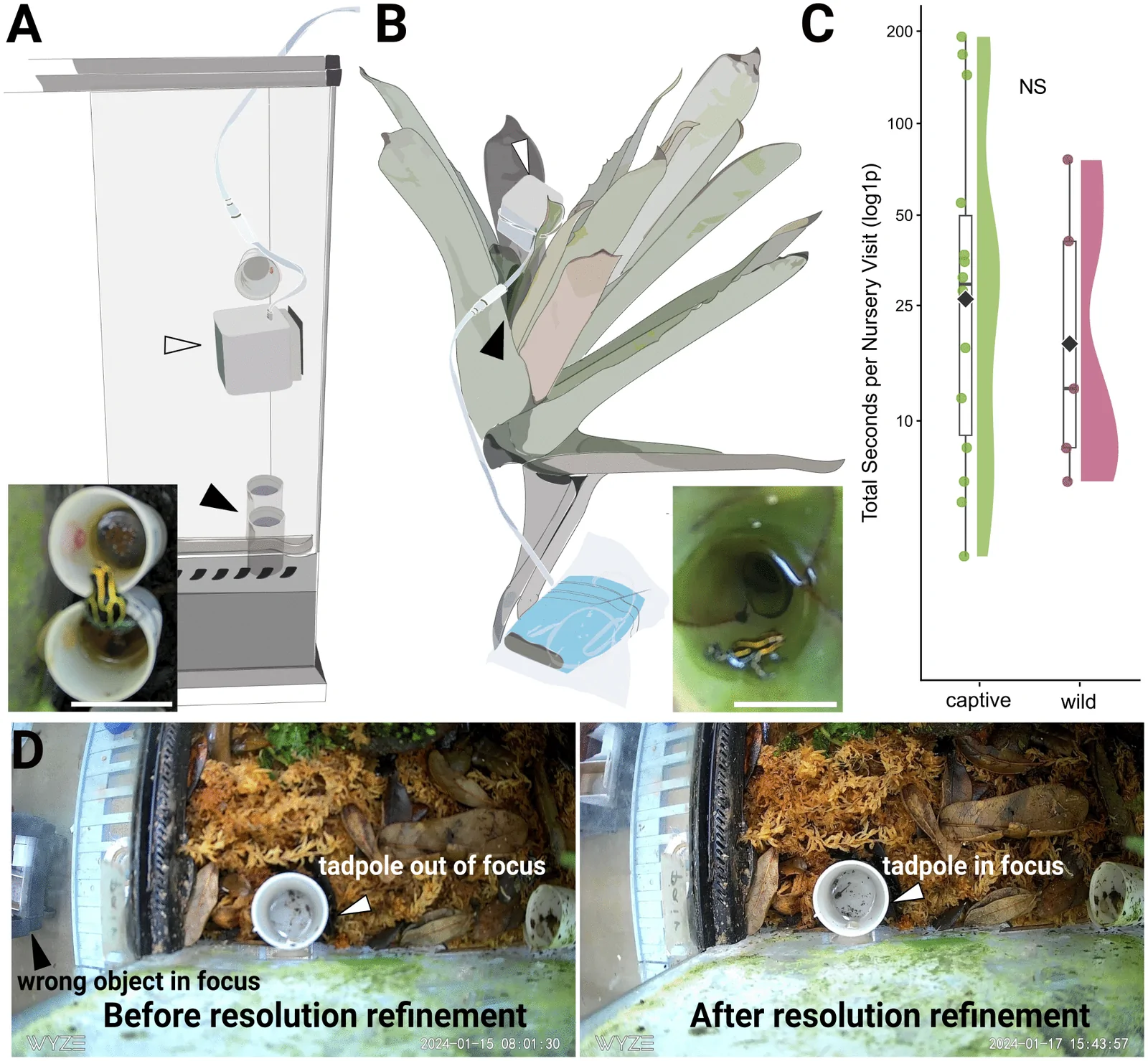
Camera installations in lab vivaria and in the field. **(A)** In lab vivaria, velcro was used to fix cameras above the desired focal subject, allowing for easy removal of the camera to switch SD cards. For monitoring parent-offspring interactions, the camera body (inlay, white arrow) was adjusted to focus on the tadpole nursery (black arrow). The USB cables were connected to an outlet via a USB charging station. Inlay pictures represent screenshots extracted from the recordings. **(B)** In their natural habitat, tadpoles grow up in water bodies formed by tightly overlapping leaf bases (black arrow, inlay) of large terrestrial plants called bromeliads. For observations, rubber bands were used to mount the cameras on bromeliad leaves above natural tadpole nurseries. The cameras (white arrows) were connected to the USB port of a power bank protected from the rain by two layers of plastic zip lock bags. **(C)** Visit length did not differ significantly between captive males or wild caregivers (purple; χ² = 0.42, *p* = 0.81, Kruskal-Wallis). **(D)** Cameras can be manually modified to improve resolution of focal sites, such as the tadpole inside of the plastic canister.

#### Sensitivity-versus-event-capture trade-off.

To quantify the cost of operating at lowest motion sensitivity (which is required to keep datasets manageable, since higher sensitivities trigger tens of thousands of recordings per day from light changes, moving vegetation, and rainfall), we positioned ten cameras above bromeliad leaf-axils after temporarily removing inhabiting tadpoles.

#### Ecological validity of laboratory-measured behavior.

We compared male parental visit duration to tadpole nurseries between captive (laboratory colony) and wild (Inselberg) *Ranitomeya variabilis* to assess whether the camera system captures comparable behavioral patterns across laboratory and field contexts.

#### Studies 1 & 2.

**Behavioral scoring**. Videos were processed and kept only when frogs entered the camera frame. The scorer marked entrance and exit times, defined as the moments at which the entire frog’s body was inside the focal canister or area. For benchmarking, observations were treated as discrete events. For the trigger-mode event-matching analysis, behavioral events were cross-matched between the two cameras using time-of-day from each camera’s internal clock, and detection was scored as TRUE if a given event appeared in a camera’s footage and FALSE otherwise. For the lab-versus-field comparison, a parental visit was defined as the entire body of an adult male entering the focal nursery, and visit duration was measured from entry to exit. For simplicity, frog attendance was measured as when the frog was present or absent for camera comparisons. In tadpole recordings, attendance was measured as the frog having the majority of its body (snout to vent) within the bromeliad or canister nursery measurements. For more qualitative behaviors not mentioned in the main text, we recorded the four qualitative and highly stereotyped behaviors described previously [25, 27]: egg care, tadpole transport, mating, pool visits (no tadpole), and pool visits (tadpole visits). Mating is when a calling male and female enter the canister together, huddling. Confirmation of mating is visually seen with the presence of an egg laid by the female. Egg care is the subsequent behavior of both sexes visiting the egg, often paired with huddling within a body’s length or on top of the egg itself. This is assumed to be parents hydrating via urination and/or guarding eggs. Tadpole transport is when a tadpole wriggles onto the adult’s back, which is visually confirmed with its body on the adult’s back. Tadpole transport concludes when the tadpole is physically off of the adult’s back. These observations are encoded in the Supplementary Data spreadsheet ‘CombinedVisits’ in the column ‘behaviors’.

### Statistical analysis

Data processing was performed in RStudio (version 4.2.3). We used non-parametric tests throughout because Shapiro-Wilk tests indicated non-normal distributions of behavioral event durations and visit lengths and because of modest sample size. We used a Wilcoxon signed-rank test to compare paired event durations between Wyze v3 and GardePro in trigger mode (matched events recorded by both cameras), and a Wilcoxon rank-sum test to compare male parental visit durations between laboratory and field settings. P-values below 0.001 are reported as p < 0.001. Figures were prepared in ggplot2 [28] and assembled in Adobe Illustrator (Adobe Illustrator 2023).

## Study 1: Results

### Technical comparison of remote monitoring systems

Across the seven systems we compared (Table 1), home security cameras showed a distinct combination of features compared with conventional camera traps. They uniformly use pixel-change detection (enabling ectotherm capture), connect to local Wi-Fi rather than cellular networks, weigh 100–400 g, and cost $35 to $100 USD. Commercial trail cameras (Reconyx, Bushnell, GardePro) operated on AA batteries for long autonomous deployments and achieved better weather resistance (IP66) but relied on passive infrared (PIR) detection, which depends on body-heat differentials and performs poorly for small ectotherms, and cost $90–460 USD. The Wyze v3 and Tapo C120 specifically combined pixel-change detection, multi-spectral night vision (both near-IR at 940 nm and far-IR at 850 nm), IP65 weather rating, and prices of $35 to $40 USD, making them the most accessible candidates for ectotherm behavioral monitoring.

### Laboratory benchmarking: In continuous mode, Wyze v3 detects more behavioral events than GardePro A3S

Over 96 h of simultaneous recording of the same focal area, Wyze v3 in continuous mode detected more behavioral observations than the GardePro A3S in trigger mode (n = 85 vs. 70 events, Fig 1C), although this difference was not statistically different between categories (χ^2^ = 1.45, df = 1, *p* = 0.23). The duration of behavior per observation also did not differ significantly between the two cameras (Wilcoxon rank-sum test, W = 3315.5, *p* = 0.20; Fig 1D). As Wyze records continuously, no event was lost to triggering failure. GardePro, by contrast, showed a triggering delay for most events: of the 68 GardePro events for which delays could be scored, 60 (88%, 95% CI: 78–95%) were missing an average 20.62 s of behavior per event compared with the continuous Wyze record (Fig 1E) and missing 324 s of behavior entirely. The cost of continuous recording was a very large fraction of empty footage, as only 1.8% of total Wyze recording time contained behavior (6,153 s with behavior vs. 333,900 s without; Fig 1F).

### In trigger mode, Wyze records significantly longer behavioral segments per event

In trigger-only mode (75% motion sensitivity) and run alongside the GardePro, the Wyze produced fewer total triggers than the GardePro (n = 47 vs. n = 60; Fig 1G). On an event-matched basis, however, the Wyze v3 detected the same behavioral event more often (38/39 events, 97.4%) than the GardePro failed to (36/39 events, 97.4%; Fig 1H). For the subset of events that both cameras successfully detected, the Wyze recorded a significantly longer interval of behavior per event than the GardePro (Wilcoxon signed-rank test with continuity correction, V = 519, *p < 0.001*; Fig 1I).

## Study 1: Discussion

Security cameras have previously been used to record behavior in frogs [29], arboreal snakes [30], fish [31], pigs [32], and chimpanzees [33]. Our laboratory results show that the Wyze v3, at approximately nine times lower cost than the GardePro, does not under-perform on any metric of behavioral capture relevant to this small ectotherm study, and outperforms the GardePro on event count (continuous mode), behavior lost per event (continuous mode), and behavior recorded per detected event (trigger mode). The single metric on which Wyze produced fewer numbers, total trigger count in trigger mode (Fig 1G), reflects fewer but longer recordings per detected event, not missed behavior, as the event-matching analysis (Fig 1H-I) shows Wyze does not miss more events than GardePro. Extending beyond the camera-comparison experiments, the Wyze system also captured detailed parental care behavior under laboratory conditions (Fig S1 in S1 File), demonstrating its utility for longitudinal studies, especially monitoring of behaviors that are rarely displayed under human observation.

Home security cameras are frequently designed to readily integrate with other software or hardware via the IFTTT (“If This, Then That”) platform [34], which enables automated workflows between programs from the moment a camera is triggered. Home security cameras can be paired with other programs, for example, with robotics for triggered behavioral manipulation ([35]), demonstrating broad cross-taxon adaptability for research. Our results establish that these cameras are suitable tools for laboratory research on small ectotherm behavior. In laboratory benchmarking, the home security camera matched or exceeded the behavioral performance of a commercial trail camera while remaining substantially less expensive.

The broader class of pixel-change home security cameras offers a viable alternative to commercial trail cameras for research and the market for such systems is rapidly expanding. Pixel-change detection is not unique to home security cameras; the more traditional Sony HDR-CX405, for instance, uses a CMOS (complementary metal-oxide-semiconductor) sensor, albeit with limited battery life. Both Wyze and Tapo cameras are lightweight, which is critical for field applications requiring long carries and extended deployments. The Tapo C120 (Table 1) is comparable in price to the Wyze v3 but has a higher IP rating and higher-resolution sensor, suggesting it may be more weather-robust and better suited to detecting small ectotherms. As the Tap C120 became commercially available after our study began, we could not benchmark it directly. However, the two-stage framework presented here, laboratory benchmarking against an established system, followed by field validation along independent hardware, sensitivity, and ecological-validity axes, provides a template for evaluating any such system. Reolink, Eufy, and Tapo/TP-Link all offer comparable pixel-change systems through globally distributed retailers, and Raspberry-Pi–based camera traps ([36]) allow further customization. Thus, our benchmarking design enables researchers to evaluate cost-effective options in advance of field work and identify the recording system best suited to their scientific question.

Combined with its lower cost, the laboratory benchmarking supports the use of the Wyze v3 over the GardePro A3S for small ectotherm recording despite Wyze’s lower weather-resistance rating (IP65 vs. IP66) and reliance on USB rather than AA power. The trigger-mode pattern of fewer but longer recordings per detected event is, for behavioral analysis, a feature rather than a deficit, as a single long recording per event preserves more behavioral context than several short fragments.

## Study 2 Results

### Hardware survival under tropical conditions

During the 1.5-month field deployment of 20 Wyze v3 units in French Guiana, 17 of 20 units remained functional to the end of the deployment despite daily rainfall of 0–30 mm and temperatures reaching 49°C at exposed rock surfaces in direct sunlight (Fig S3 in S1 File) with values up to 75 °C reported previously [23]. Three units required replacement: one camera cable was bitten by a rodent, one began producing pink-tinted recordings after one month, and one yielded little footage despite no visible damage.

Recording mode and sensitivity, once configured at the field station with Wi-Fi, were reliably preserved through offline operation. However, we identified the primary limitation to be power cycling. Each time the camera was disconnected and reconnected to a power source without Wi-Fi access, the internal timestamp reset to the factory default, and the camera reused folder names at every restart. This required SD-card replacement at every battery change to prevent file overwriting, effectively doubling the number of SD cards required relative to the number of cameras, and required filming a phone clock at startup to post-hoc reconstruct true recording times.

### Sensitivity-versus-event-capture trade-off

Of sixteen tadpole-deposition events independently verified by daily manual inspection, six were captured on camera (37.5%). Despite these limitations, the camera system still captured a range of rare and difficult to observe behaviors under natural field conditions and at night, including heterospecific intrusion, predator encounters, intraspecific aggression, and an offspring-wiping behavior not previously documented in *Ranitomeya variabilis* (Fig S2 in S1 File, Supplemental Videos S1 Video–S4 Video).

### Ecological validity of laboratory-measured behavior

We compared male parental visit duration to tadpole nurseries between captive (laboratory colony) and wild (Inselberg) *R. variabilis* to assess whether the camera system captures comparable behavioral patterns across laboratory and field contexts. Visit distributions overlapped substantially and the visit duration did not differ significantly between the two contexts (Kruskal-Wallis rank sum test, χ² = 0.42, *p* = 0.81; Fig 2C).

## Study 2: Discussion

Differences among camera systems in both event detection and recording duration can affect which ectotherm behaviors are successfully captured and documented. For example, active infrared recordings at night may be detected by nocturnal species, but this has not been systematically measured thus far and may be highly variable [37]. In our study, small poison frogs use bromeliad leaves as tadpole nurseries, which cannot support the weight of a trail camera such as the GardePro, which would therefore require additional heavy mounting equipment, such as tripods, to be carried into the field. The Wyze v3’s lower missed-event rate and longer recorded duration per detected event, as established in the laboratory benchmarking, are directly relevant to monitoring poison frog behavior in the field. Many nursery visits are short in duration and could be missed entirely by a camera with a shorter trigger-to-recording window. Conversely, longer recording durations may improve the detection and documentation of more prolonged behaviors that could otherwise be truncated, such as heterospecific intrusions or the previously undescribed offspring-wiping event.

The hardware-survival rate (17 of 20 units over 1.5 months) was consistent with the camera’s intended outdoor home-security use. It exceeded our expectations given its substantially lower acquisition cost, as we had anticipated a higher failure rate under tropical field conditions. This supports its applicability to tropical deployments.

The choice between continuous and motion-triggered modes is the most consequential decision for deployment. At lowest sensitivity, the system effectively reduces false triggers and data volume but misses a substantial fraction of true behavioral events (capturing 6 of 16 independently verified deposition events). For quantitative behavioral counts under field conditions, continuous mode is therefore preferable to low-sensitivity triggering. Nonetheless, the capture of undescribed behavior illustrated that even low-sensitivity deployments can generate novel ecological and behavioral insights, demonstrating the broader utility of the system for cost-effective field-based behavioral studies in remote or extreme weather conditions. We therefore recommend continuous mode, potentially supplemented with post-hoc AI-assisted filtering, where quantitative behavioral inference is required and low-sensitivity motion-triggered mode where qualitative or complementary observations suffice. As recording mode and sensitivity, once configured at the field station with Wi-Fi, were reliably preserved through offline operation, these settings must be chosen before deployment rather than directly in the field.

Many behavioral studies on ectotherms are conducted under laboratory conditions without evaluating whether the recorded behaviors also occur under natural conditions. At the same time, researchers working in the Global South often rely primarily on field observations because extensive captive breeding facilities are not always available and because some species will not breed readily in captivity. The absence of a significant difference in male parental visit duration between captive and wild *R. variabilis* indicates that behavior monitored with the Wyze v3 under laboratory conditions reflects behavior expressed in the wild, rather than artifacts of captivity, supporting the application of this camera system across both captive and field study settings. Together, these results demonstrate that low-cost camera systems can bridge laboratory and field observations, validating each context against the other.

However, the Wyze system also showed several important limitations that should be considered before deployment. First, in many countries the newer Wyze v4 is more readily available than the v3 and offers improved image resolution and focus at a higher price and with the same constraints for offline use. Notably, full-length video recording and uninterrupted back-to-back event capture now require a subscription service, limiting the affordability of large-scale field research deployments. Second, the practical cost of continuous-mode or high-sensitivity operation in the field is data management as recordings are automatically organized into multiple nested folders that reuse identifiers across power cycles, and managing large datasets necessitates substantial time for sorting, renaming, and ensuring chronological accuracy across devices. Some ways to circumvent this challenge include the use of computer vision and deep learning for data extraction, which has been an active area of research in machine learning [38]. For example, vision language models, such as AVA, can triage meaningful events from false positives in long or continuous data streams [39]. Likewise, open-source AI models like MegaDetector [40] are specifically designed to detect animals in camera-traps with high accuracy for downstream processing. On balance, the Wyze v3 remained a cost-effective and field-capable choice for behavioral monitoring of ectotherms. Its principal limitations, including subscription dependence in newer models and the burden of large-scale data management, are increasingly tractable as both alternative home security systems and AI-assisted video analyses mature.

## Conclusion

Our central motivation for this work is affordability and access to necessary scientific tools. Ectotherms constitute more than 99% of species diversity [41, 42], and much of that diversity is in the Global South, where research takes place within a context of historical and current colonialism and economic exploitation [43, 44] and where differences in science funding limit which questions can be asked [45]. Affordable remote monitoring is especially critical where ectotherm biodiversity is greatest and yet equipment budgets are most constrained. While the Wyze v3/v4 is currently shipped directly only within the United States and Canada and import costs can substantially increase the final price, we present it primarily as a proof of concept that home security cameras are viable low-cost alternatives to commercial trail cameras. Comparable systems are available through globally distributed retailers. An additional barrier to the broader use of home security cameras in research is language accessibility: the Wyze app interface and setup materials are primarily available in English. To reduce this limitation, we generated Spanish translations of the Wyze v3 app and quick-start guides, available via GitHub and Protocols.io (dx.doi.org/10.17504/protocols.io.dm6gp3p38vzp/v1), to help counteract the disadvantages created by the dominance of English in science [46]. We encourage researchers in the Global North to participate in efforts that improve the accessibility of scientific knowledge and research tools through collaboration and shared resources.

In summary, affordable home security cameras can match or surpass commercial trail cameras for image and video quality, detection methods, field of view, and motion-detection video length while being less expensive. Their application requires careful attention to sensitivity settings, power logistics, and data management, but the two-stage evaluation we present shows that they represent a substantial expansion of what is affordable for ectotherm behavior research, particularly in regions where the world’s ectotherm biodiversity is concentrated.

## Supporting information

S1 File Supplementary figures. Supplementary figures and information on behavior and microhabitat.
(PDF)

S1 Video Footage of heterospecific intrusion. (MP4)

S2 Video Footage of predator encounter with spider. (MP4)

S3 Video Footage of intraspecific tadpole aggression. (MP4)

S4 Video Footage of offspring-wiping behavior in *R. variabilis.* (MP4)

S2 File Behavior Data. This file contains parental behavior data.
(XLSX)

S3 File Behavior Data. This file contains perch behavior data.
(XLSX)

S4 File Analysis Code. This file contains R code used for analyses.
(R)
