# Supplementary material for: Home security cameras as a tool for behavior observations and science affordability
Source: PLoS One. 2026 Oct 26;21(10):e0360298. doi: 10.1371/journal.pone.0360298 (PMC13704821; doi:10.1371/journal.pone.0360298)
Supplement: Supplementary file 1 [file pone.0360298.s001.pdf]

Home security cameras as a tool for behavior observations and science affordability

Goolsby & Fischer et al.

Supplementary Materials

Supplementary Figures…………………...……………………………………………...……2-4
Supplementary Video Captions…………………...…………………….…………………..……5

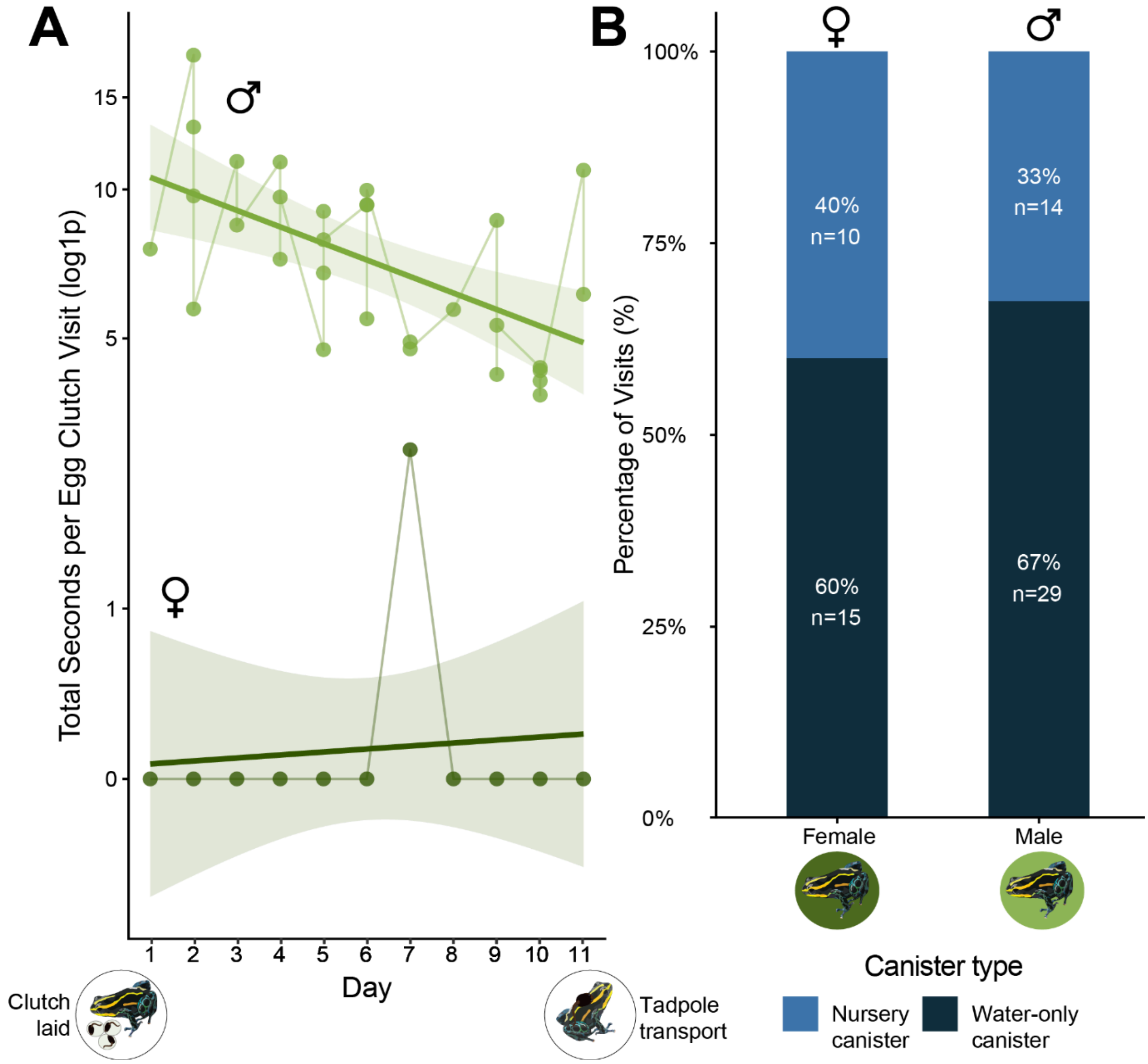

Fig S1. Comparisons of parental investment across offspring stage and in different environments. **(A)** Clutch attendance was male-only (light green), with visit duration highest immediately after egg deposition and declining as eggs matured. **(B)** Nursery visits (light blue) by male and female *R. variabilis* compared to visits to an adjacent unoccupied water canister (dark blue) during the same period.

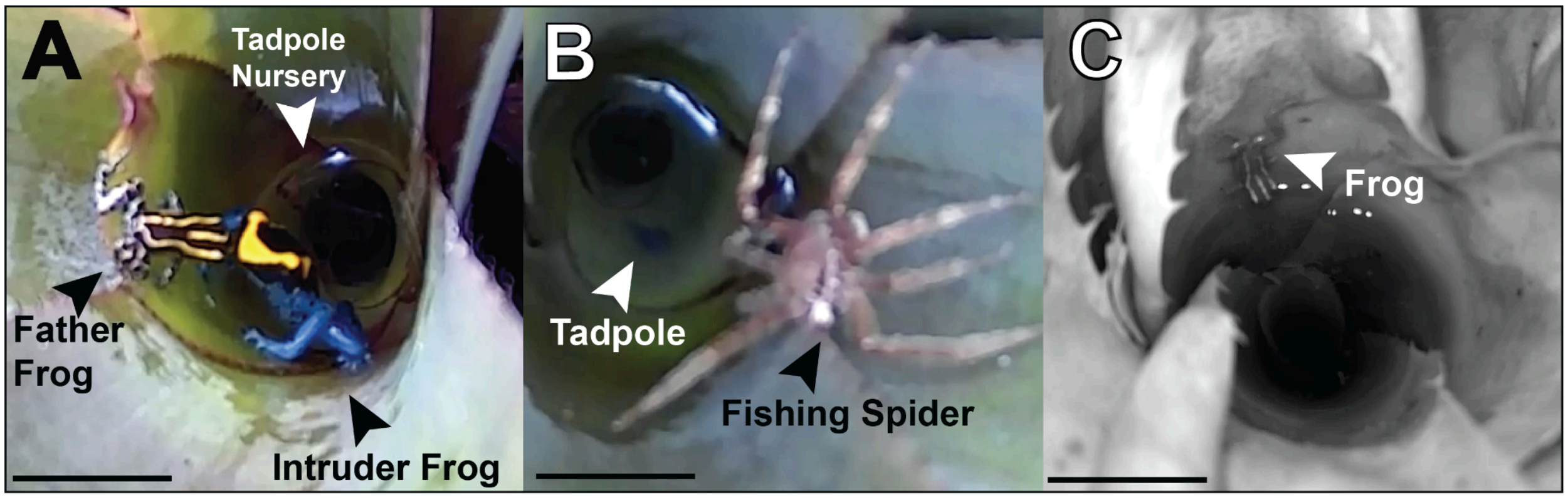

Fig. S2. Opportunistic captures of rare anuran behavioral ecology in *R. variabilis*. **(A)** Male defends his tadpole nursery from a heterospecific intruder (*Dendrobates tinctorius*). **(B)** Tadpole encounters a wandering spider (likely *Phoneutria sp*.). **(C)** Frog investigates a bromeliad pool at night. Scale bars 30 mm.

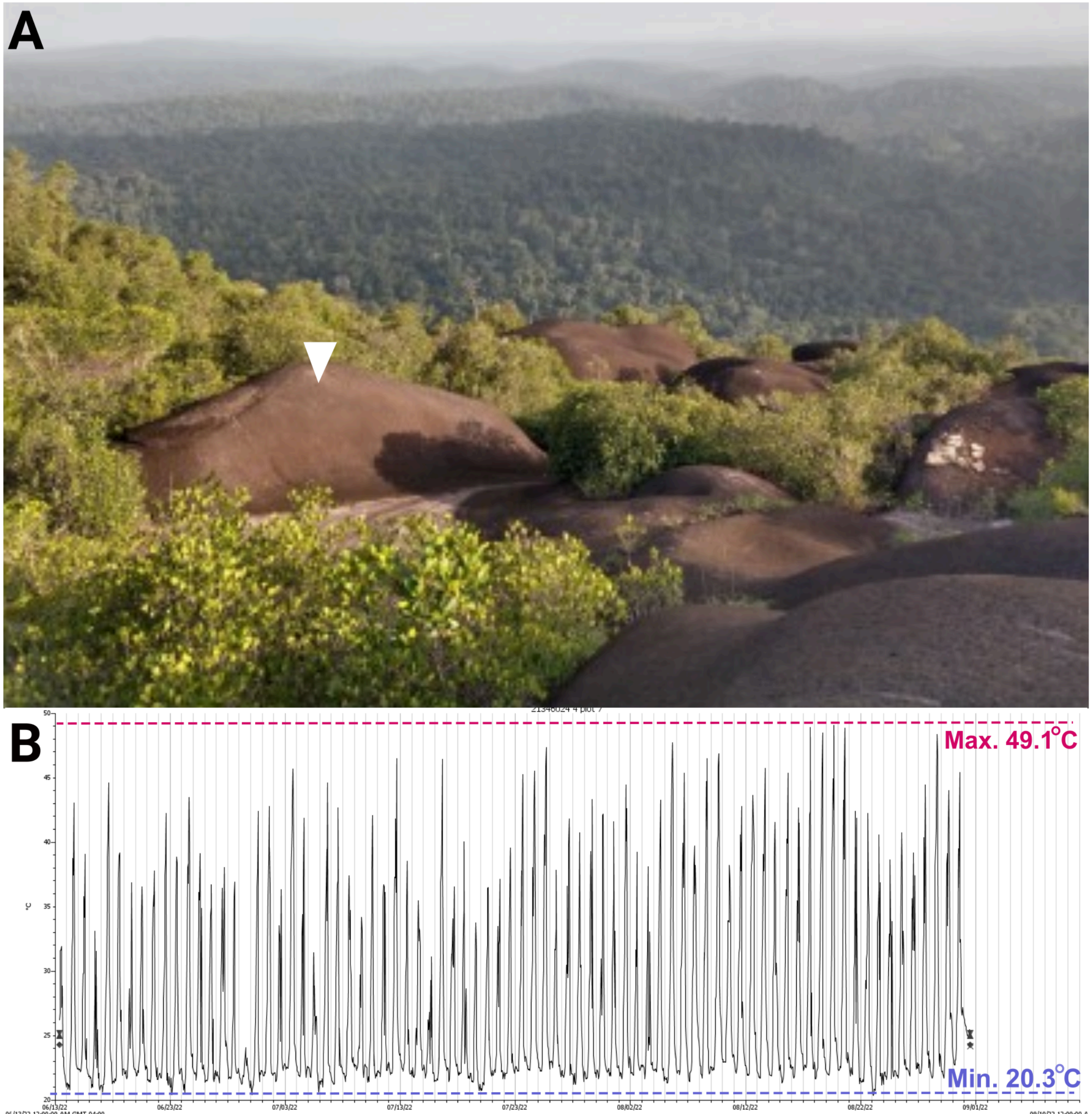

Fig. S3. Microhabitat temperatures on the Nouragues inselberg, French Guiana. (A) Summit plateau of the Nouragues Research Station Inselberg characterized by exposed granite outcrops and sparse vegetation. The white arrow indicates the position of the HOBO MX2201 temperature logger placed directly on the rock surface used by frogs for movement and activity. **(B)** Temperature profile recorded by the logger between 13 June and 31 August 2022, showing temperatures ranging from 20.33 to 49.08 °C (mean = 26.24 °C).

Supplementary Videos S1 to S4. Field and lab footage. **S1**: heterospecific intrusion. **S2**: predator encounter with spider. **S3**: intraspecific tadpole aggression. **S4**: offspring-wiping behavior in *R. variabilis*.
