# Supplementary material for: Home security cameras as a tool for behavior observations and science affordability
Source: PLoS One. 2026 Oct 26;21(10):e0360298. doi: 10.1371/journal.pone.0360298 (PMC13704821; doi:10.1371/journal.pone.0360298)
Supplement: Supplementary file 2 [file pone.0360298.s009.pdf]

Response to Review

We thank the Editor and both reviewers for their time and constructive feedback. We have substantially revised the manuscript to address the key concerns raised, particularly those of Reviewer 2, which we understand were central to the editorial decision leading to rejection. We believe these revisions substantially strengthen the manuscript and directly address the concerns that led to the original rejection. In particular, the addition of empirical benchmarking data transforms this from a proof-of-concept note into a rigorous methods study with direct performance comparisons relevant to researchers choosing between camera systems for ectotherm behavioral work. We still believe that *PLoS One* is the ideal place to publish this work and hope that you will send this manuscript for review.

Response to reviewer 1:

I have a concern over the wifi connection for phone notifications. How is this more accessible than cellular signals? Many field sites have antennae for satellite connections. Many PIR camera traps can send information over cellular signals and are used in wildlife management or for scientific data collection purposes. I would think that cellular signals can travel further distances and reach more rural or wild/undeveloped locations. Please clarify in the introduction or methods why this is an advantageous connection method.

Response: We have clarified this in the Introduction and Results sections. Cellular-enabled camera traps typically rely on Global North-based networks (e.g., AT&T, Verizon), which may have limited or no coverage in remote tropical field sites in South America, Africa, and Southeast Asia (areas with greatest ecotherm diversity). Cameras dependent on Wi-Fi like the Wyze v3 instead use locally established networks, which can be created with a portable router or phone hotspot at the field station, offering more reliable and affordable connectivity in these contexts. We have added a sentence explicitly making this distinction in the manuscript.

What exactly is pixel change? You do not describe this function of the Wyze device clearly in comparison to PIR/IR technology. This is a critical aspect of the study and I do not think you have fully explained what pixel change does for detecting movement from cold-blooded organisms.

Response: We revised the manuscript to explicitly define this in our technical comparisons – Pixel change is broadly defined as a software's comparison of pixel changes between photo or video frames, which is the basis for motion detection.

For A and C, I strongly suggest changing the colors to be more clear. Using multiple shades of green may be very difficult to discern for color-blinded individuals. Please consider using colors with greater contrast to visualize your data points.
For B, I am not sure that a pie chart is necessary to visualize the information. There is relatively little information described on the charts and it may be sufficient to just describe the pool preferences by sex in writing.

Response: We agree. All pie charts have been replaced, and this figure was substantially revised to remove any need for color to accurately interpret the image. We also confirmed accessibility by screening the figure through protanopia-type and deuteranopia–type color blindness filters.

Response to Reviewer #2:

Results should also include some type of statistical tests if possible- and there are no statistical tests done.

Response: We have added appropriate statistical tests throughout. The benchmarking comparison of event duration between Wyze and GardePro was analyzed using a Wilcoxon signed rank test with continuity correction (p=2.54E-05,V=631). We also added a Kruskal-Wallis rank sum test to compare nursery visit duration between captive and wild frogs ($\chi^2 = 0.42$, $p = 0.81$), confirming that behavioral phenotypes are consistent across environments.

I would be careful on over advertising a brand name.

Response: We have revised the manuscript to reduce brand-forward language throughout. While the Wyze v3 remains our primary case study given our empirical data, we have reframed the paper around the broader principle that home security cameras represent an accessible and affordable camera class for ectotherm behavior research. We now explicitly discuss alternative home security systems, including Google Nest and Tapo, as viable options, and emphasize that our benchmarking and validation framework can be applied to evaluate any pixel-change based camera system. The framing has been adjusted to reflect this broader scope, positioning Wyze as one example of a solution rather than the singular recommendation.

Adding a side-by-side study with trigger (vs. continuous monitoring) of multiple camera types would be a very good addition to this paper. The camera setting was on continuous so it is not apparent how this methodology is different than turning on any video recorder, as it does not appear the trigger mode was used to show it would be effectively triggered by the motion of frogs (although it likely would). Comparing 2 cameras side by side on continuous vs. trigger settings would help to evaluate this. The authors repeatedly say they evaluated Wyze cameras against other cameras but this is not accurate. Instead they present a table of specs from user manuals (see detailed comments). This manuscript would be substantially improved if they did. Adding a side-by-side study with trigger vs. continuous monitoring using the Wyze and potentially multiple other camera types would be a very good addition to this manuscript. I think it would be worthwhile for other researchers to try the method. To warrant publication in a scientific journal, I believe the study needs to be further developed to assess the camera methodology and/or the frog behavior with the manuscript revised accordingly. I appreciate being able to review this manuscript and don't want the authors to be discouraged, but hopefully encouraged to improve this and see it through.

Response: This was one of the most substantive concerns raised, and we agree it was a critical limitation of the original submission. We have now added a direct, side-by-side empirical comparison of the Wyze v3 against the GardePro A3S trail camera, recording the same behavioral arena simultaneously under both continuous (24/7) and trigger-only settings. This benchmarking study quantifies the number of behavioral events detected, the duration of recorded events, and the proportion of missed behaviors under each condition and camera type, providing the empirical performance comparison that was absent from the original manuscript. These data are now presented as a central result (Figure 2) rather than a supplementary observation.

Discuss limitations for field using protocol (need to replace powerbank every 2 days) and potential for improvement- use of marine batteries/ solar panels etc.

Response: We thank the Reviewer for this feedback. To explicitly address this, we have a new section titled "**Limitations of low-cost security cameras for field work"** to address these issues and areas for improvement.

Results and Discussion "We compared specifications, performance and availability of two monitoring systems (Reconyx [Holmen, WI, USA] and Bushnell [Overland Park, KS, USA], used in Hobbs and Brehme, 2017), two home security cameras (Wyze, used herein; Google Nest [Mountain View, CA, USA], used in Refinetti, 2020 and Hussey and LaPlante, 2024), and a manual camera system (Sony [Tokyo, Japan], used in Shen et al., 2023) used across mammals, fish, and amphibians to evaluate the suitability for lab and field studies of amphibian behavior (Table 1). We found that the home security camera Wyze v3 is on par or superior to other professional systems regarding image and video quality, detection methods, field of view and motion detection video length while being 3-18 times less expensive."

Reviewer: This should be part of methods not results on why you chose to use the cameras, perhaps with the table provided as a supplemental. It does not appear you compared performance but only spec sheets. It is not a study to compare spec sheets and claim the camera is superior without side by side testing in the lab or field. Things like actual video resolution are a function of many camera components not just software pixels. What about average battery life comparison for field use? Browning cameras are also very popular with researchers and not represented here. Adding a side-by-side study with trigger (vs. continuous monitoring) of multiple camera types would be a very good addition to this paper.

Response: We respectfully disagree that Table 1 should be in the supplementary. As we described earlier, our benchmarking and highlight of multiple suitable camera systems is worthy of keeping this table in the main article.

We first used Wyze v3 for recording and quantifying parental care behavior in laboratory vivaria. We specially tested whether there were sex differences in parental interactions with eggclutches or tadpoles in two R. variabilis families (Fig 2). Only the father showed clutch attendance, where he visited daily, with a maximum of four times in a day, over a period of 10 days.

Reviewer: Figure 2A. 1 male vs 1 female frog is not a valid test for sex differences. I suppose you could say you were able to see differences between 2 individuals and report their behavior but can't conclude anything except for how these 2 individuals behaved.

Response: We agree, and we relaxed our language here about sex differences.

We found that both the male and female preferred to visit unoccupied canister pools (Figure 2B; 60% of female visits and 67% of male visits). The number and length of visits to tadpole pools was equivalent in both parents (Fig 2C).

Reviewer: A Fishers Exact test would be appropriate for 2B. And Wilcox or Kruskill Wallis for 2C.

Response: We thank the Reviewer for this feedback. Nonparametric statistical methods were used for analyses of behavior in (now) Figs 1 & 3.

What about problems encountered with the equipment in the lab or field? Were there any? How about review of the videos? Was this a complete review or did you speed up the video to save time. It seems there was an immense amount of video to review. Consider discussing

potential for AI to help with this as a time saving measure.

Response: We thank the Reviewer for this feedback. To address this, we dedicated Figure 1 to illuminating potential issues with laboratory studies, and dedicated a section titled "**Limitations of low-cost security cameras for field work"** to address field-related issues and areas for improvement. We also explicitly discuss the immense amount of videos and that this is potentially costly of an experimenter's time.
