# Supplementary material for: Home security cameras as a tool for behavior observations and science affordability
Source: PLoS One. 2026 Oct 26;21(10):e0360298. doi: 10.1371/journal.pone.0360298 (PMC13704821; doi:10.1371/journal.pone.0360298)
Supplement: Supplementary file 3 [file pone.0360298.s010.docx]

**Response to Review**

We thank you and both reviewers for the constructive and detailed feedback. We have substantially revised the manuscript in line with the editorial and reviewer comments.

The principal change is structural. We agree the original framing made the manuscript feel heterogeneous and have reorganized the manuscript into the standard Introduction, Materials and Methods, Results, and Discussion format requested by the Editor and Reviewer #1. In doing so, we have refocused the manuscript explicitly as a two-stage methodology evaluation throughout: (i) laboratory benchmarking of the Wyze v3 against the GardePro A3S (now Fig 1, the central result), and (ii) field validation along three independent methodological axes (hardware survival under tropical conditions, sensitivity-versus-event-capture trade-off, and ecological validity of laboratory-measured behavior; now Fig 2). Behavioral observations not directly relevant to technical validation (sex differences in caregivers, canister preferences, opportunistic rare-behavior captures, *Cruziohyla craspedopus* observations) have been moved to Supporting Information. This reframing addresses the concern that the original manuscript read as "two completely different papers woven together".

We respond to each point below. Reviewer text is shown in italics; our responses follow each point. Page and line numbers refer to the revised manuscript with tracked changes.

**Response to the Editor**

*"I agree with the reviewers that the current organization of this manuscript is confusing. I believe it would greatly benefit from following a standard intro, materials and methods, results, and discussion format. The current paper also reads like two completely different papers woven together."*

**Response:** We agree on the structural point and have fully reorganized the manuscript into Introduction, Materials and Methods, Results, and Discussion. On the substantive concern about "two papers woven together," we have addressed this by reframing the field work as a methodological validation stage (along hardware, sensitivity, and ecological-validity axes) rather than as a separate behavioral case study. The Introduction now explicitly states that two complementary lines of evidence are required to establish a camera system for ectotherm behavioral research: laboratory benchmarking against an established system, and field validation under real-world conditions. The remainder of the manuscript follows this two-stage logic. The senior author has been closely involved in this revision.

*"Figure 1 contains the central results of the study. The materials, methods and results related to this are the only part of the study that needs to be described in detail. Most of the results described in figures 2-4 can be moved to the supplement, since the only point that they support is that the Wyze 3 can be used to capture a wide variety of behavioral data. The details of the behavioral data captured don't really matter in the context of the central question."*

**Response:** We have moved the behavioral-illustration content to the Supporting Information, in line with the Editor's recommendation. Original Fig 3 panels showing sex differences in caregivers and canister preferences are now S1 Fig (parental investment across offspring stage and environments). Original Fig 4 (opportunistic captures of rare behaviors) is now S2 Fig, and original Fig 5 (night recordings of *Cruziohyla craspedopus*) is now S4 Fig. We have retained two figures from the original manuscript in the main text in revised form, as they are essential to the field validation rather than illustrative of behavioral diversity: revised Fig 2 documents the field installation, the close-range refocus modification (hardware-survival evidence; this was original Fig 2), and the lab-versus-field comparison of male *R. variabilis* parental visit duration as the ecological-validity test (Fig 2C), simplified to two groups (captive males vs. wild males). Fig 1 (laboratory benchmarking) remains the primary result of the study.

*"The materials, methods, and results related to the frog study system need to be much more clearly distinguished from the materials, methods, and results related to testing whether the Wyze 3 shows similar effectiveness to a more expensive system. I would suggest creating a box that describes the frog study-system used for proof-of-concept analyses, to separate this text out from the material related to the central questions."*

**Response:** We have added a dedicated "Anuran study systems" subsection to Materials and Methods, which compactly describes the three frog species used: *Dendrobates tinctorius* as the focal species for laboratory benchmarking, *Ranitomeya variabilis* as the focal species for field validation, and *Cruziohyla craspedopus* for illustrative nocturnal observations in Supporting Information. The Materials and Methods of the main text is now organized around the two-stage evaluation: Camera systems compared, Laboratory benchmarking, Field validation (with the three validation criteria stated explicitly), Behavioral scoring, Statistical analysis, Camera setup, and Anuran study systems. This separates the methods directly required for the camera evaluation from the study-system descriptions, which are contained in the Anuran study systems subsection.

*"The results in figure 1 also need to be made much clearer. For example, it is not immediately obvious that most of them differed significantly. I would suggest that you label the sets of observations that are significantly different using standard notation (e.g., an asterisk), with the test statistics and P-values from analyses also included in each section of the figure."*

**Response:** We have revised Fig 1 accordingly. Each panel now displays the relevant significance marker directly above the compared groups (NS for not significant; *** for p < 0.001), with the test statistic and exact p-value printed in the panel (panel D: Wilcoxon rank-sum test, NS; panel I: Wilcoxon signed-rank test with continuity correction, V = 631, p < 0.001). The figure legend lists test details for each panel. Following Reviewer #1, p-values are reported in italics, and values below 0.001 are reported as *p < 0.001* rather than in exponential form (the value originally reported as p = 2.54E-05).

*"You also need to explain much more directly whether and why these results support the use of the Wyze 3 over the GardePro. It appears that the Wyze 3 often collected data that was significantly different from the GardePro. However, in most of those cases it actually collected more data, with the exception of the observations in Figure 1G. Using a standard format where (Results) you describe the results and then (Discussion) explain to us what conclusions you draw from the results would likely help with this."*

**Response:** We have added a dedicated Discussion paragraph that explicitly addresses this. We state on which axes the Wyze outperforms GardePro (event count in continuous mode; behavior lost per event; behavior recorded per detected event in trigger mode) and explicitly resolve the apparent inconsistency in Fig 1G: although the Wyze produces fewer triggers in trigger mode, the event-matched analysis in Fig 1H to 1I shows that this corresponds to fewer-but-longer recordings, not to missed events. Combined with the roughly nine-fold lower unit cost, we interpret the result as supporting Wyze preference for laboratory and small-scale field studies of small ectotherms. We also state the two axes on which Wyze is not preferred (IP65 vs. IP66 weather rating; USB vs. AA power).

*"One small point is that on the title page, the same symbol is used for the corresponding author and shared lead authorship."*

**Response:** We corrected this issue. Shared first authorship is now denoted by the dagger symbol and the corresponding author by an asterisk, with separate footnotes.

**Response to Journal Requirements**

*"Please ensure that your manuscript meets PLOS ONE's style requirements..."*

**Response:** The manuscript has been reformatted to PLOS ONE templates for the title page, main body, and figure legends.

*"In your Methods section, please provide additional information regarding the experiments involving animals and ensure you have included details on (1) methods of sacrifice, (2) methods of anesthesia and/or analgesia, and (3) efforts to alleviate suffering."*

**Response:** We have added the following statement to the Camera setup and modifications methods subsection: "No animals were sacrificed or anesthetized in this study; cameras recorded freely behaving animals in their home enclosures and in natural habitats, and no procedures requiring anesthesia or analgesia were performed."

*"We note that the grant information you provided in the 'Funding Information' and 'Financial Disclosure' sections do not match."*

**Response:** This has been corrected on the submission portal.

*"Please state what role the funders took in the study."*

**Response:** We have added the requested statement: "The funders had no role in study design, data collection and analysis, decision to publish, or preparation of the manuscript."

*"We note that Figures 1 and 2 in your submission contain copyrighted images."*

**Response:** Figure 1, does not contain any copyrighted images. In Figure 2, we took pictures of the equipment in our lab rather than using copyrighted images. See the original photo below:

*"Please include captions for your Supporting Information files at the end of your manuscript, and update any in-text citations to match accordingly."*

**Response:** Supporting Information captions for S1 to S4 Figs and S1 to S6 Videos are now provided at the end of the manuscript. All in-text references have been updated to the new S-numbering.

**Response to Reviewer #1**

*"The introduction is very brief and does not describe the importance of your case study. Why did you choose this species and behavior? What were your predictions and aims for this study?"*

**Response:** We have expanded the Introduction. The final paragraph now states the three specific benchmarking questions we ask (event count, recording duration per event, missed-event rate) and the three field-validation criteria (hardware survival, sensitivity-versus-capture trade-off, ecological validity). *Ranitomeya variabilis* was selected for field validation because its parental behavior involves transient, hard-to-observe events occurring at small spatial scales, which is exactly the use case home security cameras would need to capture, and because its population at Inselberg, French Guiana, allowed paired laboratory and field deployment under extreme environmental conditions. This rationale is now stated explicitly in the Field validation subsection of Materials and Methods.

*"I understand that the PLOS ONE submission guidelines do not state that the materials and methods section needs to come before results and discussion, but these sections would benefit from a restructured organization that is smooth and easy to follow."*

**Response:** We agree. The manuscript has been restructured into separate Introduction, Materials and Methods, Results, and Discussion sections. Results are now strictly limited to what we observed; interpretation, mechanism, and recommendations are deferred to Discussion.

*"Everything from the beginning of the results and discussion section to line 122 on page 9 could be described as methods rather than results or discussion."*

**Response:** We agree and have changed this in the revised version. Camera class comparison criteria, benchmarking design (96 h periods, sensitivity settings, focal area), field-validation criteria, behavioral scoring rules, and the rationale for non-parametric tests are now all in Materials and Methods (subsections: Camera systems compared, Laboratory benchmarking, Field validation, Behavioral scoring, Statistical analysis). The Results section opens with the technical comparison (Table 1) and proceeds to the laboratory benchmarking and then the three field-validation results in sequence.

*"I specifically focus on the paragraph starting on line 214 as an example of improving the overall structure of this study. In one paragraph, you describe your methods of measurement, report descriptive and analyzed statistics, and then interpret these results claiming that you have a novel finding on R. variabilis. While I appreciate the concise nature of such a paragraph, I lack the context as to why you set your methods this way, why you chose this test, and what the importance of this finding is to the species or goals of investigating this species."*

**Response:** That paragraph has been decomposed across the new structure: (i) the methodological design (paired nursery and adjacent water pool; daily monitoring; lab vs. field comparison) is now in the Field validation subsection of Materials and Methods; (ii) the descriptive statistics and the Kruskal-Wallis rank sum test result are reported in Results under "Field validation 3: ecological validity of laboratory-measured behavior" and displayed in Fig 2C; (iii) the interpretation is reported in Discussion, now reframed as a methodological result (laboratory measurements generalize to natural conditions) rather than a behavioral finding about the species. The original behavioral-finding content (sex differences in caregivers; canister preferences) has been moved to S1 Fig as it is not central to the camera validation.

*"For your statistical reports, please clarify why you chose each non parametric test as a best fit for your data."*

**Response:** We have added a Statistical analysis subsection to Materials and Methods explaining the choice: non-parametric tests were used because Shapiro-Wilk tests indicated non-normality of the distributions of behavioral event durations and visit lengths, and because sample sizes were modest. For paired event-duration comparisons between Wyze and GardePro in trigger mode, the Wilcoxon signed-rank test with continuity correction was used because data were paired by event. For the lab-versus-field comparison of male parental visit duration, a Kruskal-Wallis rank sum test was used. The choice of test for each comparison is also restated at the point of use in Results and in the figure legends.

*"Additionally, please italicize your p values and I strongly recommend reporting all values below 0.001 as <0.001. On Line 158, you report a p value left in exponential form, which makes it less accessible and clear."*

**Response:** All *p* values are now italicized throughout the text, figure legends, and tables. The value previously reported as *p* = 2.54E-05 is now reported as *p < 0.001*. Test statistics and the abbreviation NS are italicized where used.

**Response to Reviewer #2**

*"One issue I noticed was that a reviewer requested statistical tests for comparisons between two cameras; the authors said that they added tests, but I saw only one test in the whole article."*

**Response:** Every comparison reported in Results is now accompanied by an explicit test, test statistic, p-value, and reference to the Methods subsection in which the test choice is justified. For Fig 1, the comparisons reported are: number of observations in continuous mode (descriptive, Fig 1C); observation length in continuous mode (Wilcoxon rank-sum test, W = 3315.5, NS, Fig 1D); seconds lost per recording in continuous mode (descriptive mean, Fig 1E); proportion of recording time with behavior (descriptive, Fig 1F); number of triggers in trigger mode (descriptive, Fig 1G); event-matched detection concordance (descriptive, Fig 1H); and event-matched behavior duration (Wilcoxon signed-rank test with continuity correction, V = 631, p < 0.001, Fig 1I). For the field validation ecological-validity test (Fig 2C), male parental visit duration was compared between captive and wild *R. variabilis* using a Kruskal-Wallis rank sum test (χ² = 0.42, p = 0.81, NS).

*"The article is interesting and is well written, although the style is heterogeneous. The article is partially a methodology study (of using simple cameras for the study of animal behavior) and partially a field report of frog behavior (Dendrobates tinctorius and Ranitomeya variabilis). For a methodology article, the description of frog behavior could be greatly shortened."*

**Response:** We agree the original framing was heterogeneous. We have restructured the manuscript as a two-stage methodology evaluation throughout: laboratory benchmarking (stage 1) followed by field validation (stage 2). The field validation is now explicitly framed as testing three methodological criteria (hardware survival, sensitivity-versus-capture trade-off, ecological validity of laboratory measurements) rather than as a behavioral study of *R. variabilis*. The behavioral observations not central to validation (sex differences in caregivers, canister preferences) have been moved to S1 Fig; opportunistic rare-behavior captures are now S2 Fig; and *Cruziohyla craspedopus* observations are now S4 Fig. The frog species used are now summarized compactly in an "Anuran study systems" subsection of Materials and Methods.

*"Table 1 is the heart of the manuscript and provides the main information in the article."*

**Response:** In the revised structure, Table 1 (technical comparison of seven camera systems) and Fig 1 (Wyze v3 vs. GardePro A3S benchmarking) together carry the central results. The Results section opens with Table 1 and proceeds directly to the empirical benchmarking presented in Fig 1, before turning to the field validation.

*"On page 4, line 89, the subject of the sentence is plural ('both home security cameras') but the verb is conjugated in the singular ('has')."*

**Response:** Corrected: "both home security cameras Wyze v3 and Tapo have multiple degrees of night vision capabilities..."

*"On page 5, line 114, prices are said to range from upwards of $450 USD per camera to $35 USD; although the passage is not grammatically wrong, ranges are usually reported from lower to higher."*

**Response:** Corrected. The sentence now reads "Camera prices ranged from $35 USD to upwards of $450 USD per camera."

*"On page 10, line 172, temperatures in the field are reported to range from 20.5°C to 50°C; this may be correct, but 50°C is a very high temperature; was this measured in the air (unlikely) or on the surface of an object under direct sunlight (more likely)?"*

**Response:** 50°C was a surface-temperature measurement. The temperature was recorded at exposed granite-rock surfaces in direct sunlight on the Inselberg of the Nouragues Nature Reserve. We refer to substrate temperature rather than air temperature because this measure is more biologically relevant for these frogs as they are in direct contact with the substrate, making substrate temperature a more accurate representation of the thermal conditions they experience than the temperature of the surrounding air.Even higher surface-temperatures (up to 75°C) were reported for this field site (Sarthou et al. 2009; ref 34). The text in both the Introduction (Field validation paragraph) and the Methods now clarifies this and we added a Supplementary Figure (Fig. S3) depicting the measurement location as well as the temperature profile recorded by the logger.
