# Supplementary material for: Home security cameras as a tool for behavior observations and science affordability
Source: PLoS One. 2026 Oct 26;21(10):e0360298. doi: 10.1371/journal.pone.0360298 (PMC13704821; doi:10.1371/journal.pone.0360298)
Supplement: Supplementary file 4 [file pone.0360298.s011.docx]

**Response to the reviewers**

We thank the reviewers for their thoughtful and constructive comments, which have helped us to improve the manuscript. Below, we detail the changes made in response to each comment. Below, the reviewers’ comments are shown in black, followed by our responses in blue. For the reviewer’s convenience, all lines refer to the untracked version of the manuscript. We also found a small figure 1 error, which is now corrected (Fig 1H was accidentally labeled Fig1I, and Fig 1I was accidentally labeled Fig1J).

**Reviewer #3:**

I think overall this is an excellent study and does valuable work advocating for understudied species and science accessibility. My issues with the manuscript are primarily in terms of organization. While there are marked improvements from the previous version, there are issues throughout where information appropriate for the introduction or methods section is not disclosed until the results or discussion.

In the revised version, we have addressed this comment through the reorganization described point-by-point below.

I think the introduction would benefit from more detail to provide readers with necessary context. Some statements require citations (e.g. line 40 that Global South has much of ectotherm biodiversity).

We thank the reviewer for this feedback. For clarity, we have added detail and citations, specifically the following:

[3.	Martin PC, Bateson P. Measuring behaviour: An introductory guide. Cambridge, England: Cambridge University Press; 1986.](https://paperpile.com/b/TBIVwhF5/WQO1)

[11.	Welbourne DJ, Claridge AW, Paull DJ, Lambert A. How do passive infrared triggered camera traps operate and why does it matter? Breaking down common misconceptions. Remote Sens Ecol Conserv. 2016;2: 77–83.](https://paperpile.com/b/TBIVwhF5/2bdE)

[12.	Welbourne DJ, Paull DJ, Claridge AW, Ford F. A frontier in the use of camera traps: surveying terrestrial squamate assemblages. Remote Sens Ecol Conserv. 2017;3: 133–145.](https://paperpile.com/b/TBIVwhF5/q3Mm)

[13.	Rovero F, Zimmermann F, Berzi D, Meek P. “Which camera trap type and how many do I need?” A review of camera features and study designs for a range of wildlife research applications. Hystrix-italian Journal of Mammalogy. 2013;24: 148–156.](https://paperpile.com/b/TBIVwhF5/bZbk)

[14.	Collen B, Ram M, Zamin T, McRae L. The tropical biodiversity data gap: Addressing disparity in global monitoring. Trop Conserv Sci. 2008;1: 75–88.](https://paperpile.com/b/TBIVwhF5/a1qN)

[15.	Mugerwa B, Niedballa J, Planillo A, Sheil D, Kramer-Schadt S, Wilting A. Global disparity of camera trap research allocation and defaunation risk of terrestrial mammals. Remote Sens Ecol Conserv. 2024;10: 121–136.](https://paperpile.com/b/TBIVwhF5/yXg1)

[16.	Whytock RC, Suijten T, van Deursen T, Świeżewski J, Mermiaghe H, Madamba N, et al. Real-time alerts from AI-enabled camera traps using the Iridium satellite network: a case-study in Gabon, Central Africa. Ecology. bioRxiv; 2021. Available: https://www.biorxiv.org/content/10.1101/2021.11.10.468078v1.full.pdf](https://paperpile.com/b/TBIVwhF5/SKAF)

[17.	Radke RJ, Andra S, Al-Kofahi O, Roysam B. Image change detection algorithms: a systematic survey. IEEE Trans Image Process. 2005;14: 294–307.](https://paperpile.com/b/TBIVwhF5/0OTL)

[37.	Meek PD, Ballard G-A, Fleming PJS, Schaefer M, Williams W, Falzon G. Camera traps can be heard and seen by animals. PLoS One. 2014;9: e110832.](https://paperpile.com/b/TBIVwhF5/coQV)

The paragraphs describing the differences between camera traps and home security cameras (lines 46-60) are lacking some potentially relevant details, such as the difference between how camera systems generally record daytime and nighttime videos.

This has been expanded upon and clarified in lines 39-44 and 54-59.

If there is some form of IR that can detect ectotherms, this should be mentioned in the introduction.

There have been successful PIR recordings in mostly larger squamates such as crocodilians. In smaller squamates, PIR recording has been successfully done with a modified version of PIR called camera overhead augmented temperature (COAT) in Welbourne *et al.* 2013 (citation 12 above reviews this) which leverages the difference of squamate surface heat (similar to environment) compared to a cork background. Lines 41-43 hope to clarify this point.

While it makes sense that the introduction would focus on the differences between the two camera sources, I think the focal species and behaviors of interest should be described in the introduction, briefly, and in the context of what observational challenges would be directly improved by the results of this study. This information is present in Field validation subsection of methods, currently.

In response to this concern, a new paragraph was added to the end of the Introduction describing both study species (*D. tinctorius* and *R. variabilis*) and explaining their respective advantages for addressing the research question.

The author’s introduction should end with their hypotheses about how the two camera systems will perform in the lab and wild.

A new closing paragraph was added to the introduction stating the hypotheses of this work.

The methods section has discrete subheaders, which is great and a marked improvement in organization from the previous version. However, I think some of them would benefit from being expanded and reorganized.

We addressed this point via the restructuring described below.

The authors might consider having a multistudy organization such that the camera systems comparison and laboratory study is reported in full (methods, results, discussion) before reporting on the wild study (with another methods, results, and discussion section), where the emphasis can remain on how the camera systems differed in terms of accurate event recording.

We thank the reviewer for this helpful suggestion. Following this recommendation, we reorganized the manuscript using a multi-study structure. The manuscript is now divided into Study 1: Camera Comparison and Laboratory Benchmarking and Study 2: Field Validation, each with its own Results and Discussion sections. We retained a shared Materials and Methods section because both studies rely on common approaches for behavioral coding and statistical analyses. However, this section has been subdivided into methods common to both studies, methods and study system specific to Study 1, and methods and study system specific to Study 2.

The observations of C. craspedopus, unless they reveal something critical about Wayze’ nighttime capacities not captured in the other two experiments, may be better off saved for a future manuscript.

The *C. craspedopus* study system and all associated body text have been removed.

If this reorganization suggestion is pursued, anuran study systems can be separated and organized with the corresponding experiment. Otherwise, I think it would provide better narrative flow to place that section earlier in the methods, before behavioral coding.

We followed the reviewer’s advice and introduced *D. tinctorius* under Study 1 ("Anuran study system in the laboratory") and *R. variabilis* under Study 2 ("Anuran study system in the field").

Environmental factors (brightness, humidity, etc) seem relevant in this case and could be included when describing the terrarium.

We followed the reviewer’s advice, and this has been added under Study 1 (“Anuran study system in the laboratory”).

Behavioral scoring would benefit from being accompanied by an ethogram of the behaviors studied.

For simplicity, frog attendance was measured as when the frog was present or absent for camera comparisons. In tadpole recordings, attendance was measured as the frog having the majority of its body (snout to vent) within the bromeliad or canister nursery measurements. For more qualitative behaviors not mentioned in the main text, we recorded the four qualitative and highly stereotyped behaviors described previously in Brown et al., 2008: egg care, tadpole transport, mating, pool visits (no tadpole), and pool visits (tadpole visits). Mating is when a calling male and female enter the canister together, huddling. Confirmation of mating is visually seen with the presence of an egg laid by the female. Egg care is the subsequent behavior of both sexes visiting the egg, often paired with huddling within a body’s length or on top of the egg itself. This is assumed to be parents hydrating via urination and/or guarding eggs. Tadpole transport is when a tadpole wriggles onto the adult’s back, which is visually confirmed with its body on the adult’s back. Tadpole transport concludes when the tadpole is physically off of the adult’s back. These observations are encoded in the Supplementary Data spreadsheet ‘CombinedVisits’ in the column ‘behaviors’.

The amount of footage and total number of events (and total count per behavior) recorded by each camera system should be included.

We described this in passing in ‘Laboratory benchmarking: Wyze v3 detects more behavioral events than GardePro A3S in continuous mode’, but we agree with reviewers that this section would benefit from explicit descriptions of the footage and events recorded and therefore has been added.

Finally, the "Statistical analysis" section should be the final section of methods and precede the results.

Statistical analysis is now the last general Methods subsection (under "Studies 1 & 2"), immediately before the Results.

Given that no statistical analysis is necessary to describe the differences between the camera systems, Table 1 and lines 193-203 would fit better in the beginning of the methods section, under the "Camera systems compared" subheader (line 76), where the content of that table is first discussed.

We did not address this comment, as we respectfully disagree with the suggested change. Descriptive results are commonly reported without statistical analysis, for example in case studies, systematic reviews, or methodological and technical comparisons. The table summarizes the outcome of our systematic comparison of the camera systems and therefore represents a result of Study 1 rather than a description of the methods used. The comparison also informed the choice of camera model and will be useful to researchers selecting a camera system that best matches their study question. Accordingly, this comparison was considered one of the most important components of the study by other reviewers. We therefore consider Table 1 and the corresponding text better suited to the Results section.

In the paragraph of lines 211-220, the authors argue that "Wyze v3 detects more behavioral events than GardePro A3S in continuous mode" however I don’t see a p value to support this statement. If the loss of 21 seconds per recording led to statistically significant differences in numbers of events recorded, the test determining that should be reported.

This has been clarified to soften the language of this claim, given the count difference was statistically insignificant. However, it is difficult to run a statistical analysis for recording loss with Wyze in continuous mode vs GardePro, as our a priori expectations of data loss cannot be 50/50 with one camera recording 24/7 with 0 seconds of data loss. Thus, we added a comparison of the proportion of data lost (60/68 of events detected by GardePro experienced data loss; 88%, 95% CI 78-95%).

Along that line of thought, figure 1C and 1D would benefit from denoting significance level between camera types.

We thank the reviewer for this suggestion and have added “NS” to both panels to specify non significance.

Lines 228-237 are a lovely summary of the preceding results and are better suited as the first paragraph of the discussion.

This summary paragraph was merged with a sentence about the previous use of security cameras and now opens the Study 1 Discussion.

Lines 257-306 return to the heterogenous organization and are a mixture of information appropriate for methods, results, and discussion. The authors should try to keep descriptions of field sites and procedures in the methods, limit the results to the outcomes of statistical analysis (or demographics of the dataset), and save the majority of interpretations or any mentions of alternative solutions to limitations for the discussion.

We addressed the reviewers' concern by restructuring the manuscript. Specifically, field-site and procedural descriptions were moved into the Methods for Study 2, Results now report outcomes only. The interpretation of the results has been moved into the Study 2 Discussion, alongside the limitations.

The discussion in lines 388-404 is excellent and demonstrates the importance of this work.

We thank the reviewer and moved this general part of the discussion to a new “Conclusions” part with preserved verbatim.

Information about camera systems differences might benefit from being described in the context of the specific frog behaviors observed by the study.

We added an introductory paragraph to the discussion of the field study. In the revised version, it now reads:

“Differences among camera systems in both event detection and recording duration can affect which ectotherm behaviors are successfully captured and documented. Tadpole nurseries of small poison frogs, in this case bromeliad leaves, cannot support the weight of a trail camera such as the GardePro, which would therefore require additional heavy mounting equipment, such as tripods, to be carried into the field. The Wyze v3's lower missed-event rate and longer recorded duration per detected event, as established in the laboratory benchmarking, are directly relevant to monitoring poison frog behavior in the field. Many nursery visits are short in duration and could be missed entirely by a camera with a shorter trigger-to-recording window. Conversely, longer recording durations may improve the detection and documentation of more prolonged behaviors that could otherwise be truncated, such as heterospecific intrusions or the previously undescribed offspring-wiping event.”

Typographical or grammatical errors:

line 35: "bth" rather than "both"

line 78: "...the suitability" it is more appropriate to use "their suitability" in this case

lines 108-113: This is a complex, multi-clause sentence. Consider breaking up into two or three sentences. Also, "air-to-surface temperature" is a bit unclear; is this the range of both air and surface temperatures? Consider reporting each individually.

line 195: The colon can be replaced by a period. As it stands, 194-197 is another run on sentence.

line 216-217: Consider breaking this up into two sentences (replaced "but" with "however")

line 286: difficult-to-observe; I think the hyphens are unnecessary

line 333: unnecessary comma

line 347: "identifying" should be "identify"

line 372: Again, consider replacing colon with period and making two separate sentences

All typographical errors mentioned by reviewer 3 were corrected as suggested.

**Reviewer #2**: answered “Yes” to all assessment questions and provided no specific comments requiring a response.
