# Supplementary material for: Home security cameras as a tool for behavior observations and science affordability
Source: PLoS One. 2026 Oct 26;21(10):e0360298. doi: 10.1371/journal.pone.0360298 (PMC13704821; doi:10.1371/journal.pone.0360298)
Supplement: Peer review history

# Author comment: Author response to Decision Letter 0

**DOI:** 10.1371/journal.pone.0360298.r001

22 Mar 2026

Attachment Submitted filename: PLOS_responsetoreview.pdf

# Peer review: Decision Letter 0

**DOI:** 10.1371/journal.pone.0360298.r002

**Authors:** Patrick Stephens

25 Apr 2026

Dear Dr. O'Connell,

Thank you for submitting your manuscript to PLOS ONE. After careful consideration, we feel that it has merit but does not fully meet PLOS ONE’s publication criteria as it currently stands. Therefore, we invite you to submit a revised version of the manuscript that addresses the points raised during the review process.

I agree with the reviewers that the current organization of this manuscript is confusing.  I believe it would greatly benefit from following a standard intro, materials and methods, results, and discussion format.  The current paper also reads like two completely different papers woven together.  I would suggest that you solicit more input from the senior author to help revise the manuscript for improved organization and clarity.

Figure 1 contains the central results of the study.  The materials, methods and results related to this are the only part of the study that needs to be described in detail.  Most of the results described in figures 2-4 can be moved to the supplement, since the only point that they support is that the Wyze 3 can be used to capture a wide variety of behavioral data.   The details of the behavioral data captured don’t really matter in the context of the central question.

The materials, methods, and results related to the frog study system need to be much more clearly distinguished from the materials, methods, and results related to testing whether the Wyze 3 shows similar effectiveness to a more expensive system.  I would suggest creating a box that describes the frog study-system used for proof-of-concept analyses, to separate this text out from the material related to the central questions.

The results in figure 1 also need to be made much clearer.  For example, it is not immediately obvious that most of them differed significantly.  I would suggest that you  label the sets of observations that are significantly different using standard notation (e.g., an asterisk), with the test statistics and P-values from analyses also included in each section of the figure.  The other option would be to supplement the figure with a table listing each statistical analysis.  Regardless, readers shouldn’t have to dig into the text to figure out which of these sets of observations are significantly different at alpha.

You also need to explain much more directly whether and why these results support the use of the Wyze 3 over the GardePro.  It appears that the Wyze 3 often collected data that was significantly different from the GardePro.  However, in most of those cases it actually collected more data, with the exception of the observations in Figure 1G.    Using a standard format where (Results) you describe the results and then (Discussion) explain to us what conclusions you draw from the results would likely help with this.

One small point is that on the title page, the same symbol is used for the corresponding author and shared lead authorship.  Unless it is your intention to convey that there are three lead authors and three corresponding authors, you need to correct this.

Please submit your revised manuscript by Jun 09 2026 11:59PM. If you will need more time than this to complete your revisions, please reply to this message or contact the journal office at plosone@plos.org. When you're ready to submit your revision, log on to https://www.editorialmanager.com/pone/ and select the 'Submissions Needing Revision' folder to locate your manuscript file.

- A letter that responds to each point raised by the academic editor and reviewer(s). You should upload this letter as a separate file labeled 'Response to Reviewers'.
- A marked-up copy of your manuscript that highlights changes made to the original version. You should upload this as a separate file labeled 'Revised Manuscript with Track Changes'.
- An unmarked version of your revised paper without tracked changes. You should upload this as a separate file labeled 'Manuscript'.

If you would like to make changes to your financial disclosure, please include your updated statement in your cover letter. Guidelines for resubmitting your figure files are available below the reviewer comments at the end of this letter.

If applicable, we recommend that you deposit your laboratory protocols in protocols.io to enhance the reproducibility of your results. Protocols.io assigns your protocol its own identifier (DOI) so that it can be cited independently in the future. For instructions see: https://journals.plos.org/plosone/s/submission-guidelines#loc-laboratory-protocols. Additionally, PLOS ONE offers an option for publishing peer-reviewed Lab Protocol articles, which describe protocols hosted on protocols.io. Read more information on sharing protocols at https://plos.org/protocols?utm_medium=editorial-email&utm_source=authorletters&utm_campaign=protocols.

As the corresponding author, your ORCID iD is verified in the submission system and will appear in the published article. PLOS supports the use of ORCID, and we encourage all coauthors to register for an ORCID iD and use it as well. Please encourage your coauthors to verify their ORCID iD within the submission system before final acceptance, as unverified ORCID iDs will not appear in the published article. *Only*  the individual author can complete the verification step; PLOS staff *cannot*  verify ORCID iDs on behalf of authors.

We look forward to receiving your revised manuscript.

Kind regards,

Patrick R Stephens, Ph.D.

Academic Editor

PLOS One

**Journal Requirements:**

When submitting your revision, we need you to address these additional requirements.

1. Please ensure that your manuscript meets PLOS ONE's style requirements, including those for file naming. The PLOS ONE style templates can be found at

https://journals.plos.org/plosone/s/file?id=wjVg/PLOSOne_formatting_sample_main_body.pdf and

https://journals.plos.org/plosone/s/file?id=ba62/PLOSOne_formatting_sample_title_authors_affiliations.pdf

2. To comply with PLOS One submissions requirements, in your Methods section, please provide additional information regarding the experiments involving animals and ensure you have included details on (1) methods of sacrifice, (2) methods of anesthesia and/or analgesia, and (3) efforts to alleviate suffering.

3. We note that the grant information you provided in the ‘Funding Information’ and ‘Financial Disclosure’ sections do not match.

When you resubmit, please ensure that you provide the correct grant numbers for the awards you received for your study in the ‘Funding Information’ section.

4. Thank you for stating the following financial disclosure:

“This research was funded with grants from National Institutes of Health (DP2HD102042), the Rita Allen Foundation, the McKnight Foundation, Pew Charitable Trusts, and the New York Stem Cell Foundation to LAO. BCG is supported by an HHMI Gilliam Fellowship (GT15685) and a National Institutes of Health Cellular Molecular Biology Training Grant (T32GM007276). MF is supported by an Erwin Schrödinger fellowship from the FWF (J-4526B). AL is supported by a Matthew Pecot Fellowship from the McKnight Foundation. LAO is a New York Stem Cell Foundation – Robertson Investigator.”

Please state what role the funders took in the study.  If the funders had no role, please state: "The funders had no role in study design, data collection and analysis, decision to publish, or preparation of the manuscript."

If this statement is not correct you must amend it as needed.

Please include this amended Role of Funder statement in your cover letter; we will change the online submission form on your behalf.

5. We note that Figures 1 and 2 in your submission contain copyrighted images. All PLOS content is published under the Creative Commons Attribution License (CC BY 4.0), which means that the manuscript, images, and Supporting Information files will be freely available online, and any third party is permitted to access, download, copy, distribute, and use these materials in any way, even commercially, with proper attribution. For more information, see our copyright guidelines: http://journals.plos.org/plosone/s/licenses-and-copyright.

We require you to either (1) present written permission from the copyright holder to publish these figures specifically under the CC BY 4.0 license, or (2) remove the figures from your submission:

1. You may seek permission from the original copyright holder of Figures 1 and 2 to publish the content specifically under the CC BY 4.0 license.

We recommend that you contact the original copyright holder with the Content Permission Form (http://journals.plos.org/plosone/s/file?id=7c09/content-permission-form.pdf) and the following text:

“I request permission for the open-access journal PLOS ONE to publish XXX under the Creative Commons Attribution License (CCAL) CC BY 4.0 (http://creativecommons.org/licenses/by/4.0/). Please be aware that this license allows unrestricted use and distribution, even commercially, by third parties. Please reply and provide explicit written permission to publish XXX under a CC BY license and complete the attached form.”

Please upload the completed Content Permission Form or other proof of granted permissions as an "Other" file with your submission.

In the figure caption of the copyrighted figure, please include the following text: “Reprinted from [ref] under a CC BY license, with permission from [name of publisher], original copyright [original copyright year].”

2. If you are unable to obtain permission from the original copyright holder to publish these figures under the CC BY 4.0 license or if the copyright holder’s requirements are incompatible with the CC BY 4.0 license, please either i) remove the figure or ii) supply a replacement figure that complies with the CC BY 4.0 license. Please check copyright information on all replacement figures and update the figure caption with source information. If applicable, please specify in the figure caption text when a figure is similar but not identical to the original image and is therefore for illustrative purposes only.

6. Please include captions for your Supporting Information files at the end of your manuscript, and update any in-text citations to match accordingly. Please see our Supporting Information guidelines for more information: http://journals.plos.org/plosone/s/supporting-information.

7. If the reviewer comments include a recommendation to cite specific previously published works, please review and evaluate these publications to determine whether they are relevant and should be cited. There is no requirement to cite these works unless the editor has indicated otherwise.

[Note: HTML markup is below. Please do not edit.]

Reviewers' comments:

Reviewer's Responses to Questions

**Comments to the Author**

1. Is the manuscript technically sound, and do the data support the conclusions?

Reviewer #1: Partly

Reviewer #2: Yes

**********

2. Has the statistical analysis been performed appropriately and rigorously?

Reviewer #1: I Don't Know

Reviewer #2: No

**********

3. Have the authors made all data underlying the findings in their manuscript fully available?

The PLOS Data policy

Reviewer #1: Yes

Reviewer #2: Yes

**********

4. Is the manuscript presented in an intelligible fashion and written in standard English?

Reviewer #1: Yes

Reviewer #2: Yes

**********

Reviewer #1: The introduction is very brief and does not describe the importance of your case study. Why did you choose this species and behavior? What were your predictions and aims for this study?

I understand that the PLOS ONE submission guidelines do not state that the materials and methods section needs to come before results and discussion, but these sections would benefit from a restructured organization that is smooth and easy to follow. I also understand that the results and discussions sections can be combined but I recommend being much more clear about what is a reported result and what is an interpretation for your disucssion. Everything from the beginning of the results and discussion section to line 122 on page 9 could be described as methods rather than results or discussion. Further, I recommend creating a new section after reporting your results where you interpret them, rather than immediately describing the importance of them.

I specifically focus on the paragraph starting on line 214 as an example of improving the overall structure of this study. In one paragraph, you describe your methods of measurement, report descriptive and analyzed statistics, and then intepret these results clamining that you have a novel finding on R. variabilis. While I appreciate the concise nature of such a paragraph, I lack the context as to why you set your methods this way, why you chose this test, and what the importance of this finding is to the species or goals of investigating this specices.

For your statistical reports, please clarify why you chose each non parametric test as a best fit for your data. Additionally, please italicize your p values and I strongly recommend reporting all values below 0.001 as <0.001. On Line 158, you report a p value left in exponential form, which makes it less accessible and clear.

Reviewer #2: Before making comments about the contents of the manuscript, I must indicate that I did not review the original version. The authors seem to have revised the manuscript in accordance with the suggestions made by the two reviewers. One issue I noticed was that a reviewer requested statistical tests for comparisons between two cameras; the authors said that they added tests, but I saw only one test in the whole article.

The article is interesting and is well written, although the style is heterogeneous. The article is partially a methodology study (of using simple cameras for the study of animal behavior) and partially a field report of frog behavior (Dendrobates tinctorius and Ranitomeya variabilis). For a methodology article, the description of frog behavior could be greatly shortened. Table 1 is the heart of the manuscript and provides the main information in the article. The provided information is valuable and should be of assistance to many field researchers.

As a minor comment, the authors should do a final reading of the manuscript to eliminate small errors. On page 4, line 89, the subject of the sentence is plural ("both home security cameras") but the verb is conjugated in the singular ("has"). On page 5, line 114, prices are said to range from upwards of $450 USD per camera to $35 USD; although the passage is not grammatically wrong, ranges are usually reported from lower to higher. On page 10, line 172, temperatures in the field are reported to range from 20.5°C to 50°C; this may be correct, but 50°C is a very high temperature; was this measured in the air (unlikely) or on the surface of an object under direct sunlight (more likely)?

**********

what does this mean?). If published, this will include your full peer review and any attached files.

If you choose “no”, your identity will remain anonymous but your review may still be made public.

**Do you want your identity to be public for this peer review?**  For information about this choice, including consent withdrawal, please see our Privacy Policy

Reviewer #1: No

Reviewer #2: No

**********

[NOTE: If reviewer comments were submitted as an attachment file, they will be attached to this email and accessible via the submission site. Please log into your account, locate the manuscript record, and check for the action link "View Attachments". If this link does not appear, there are no attachment files.]

To ensure your figures meet our technical requirements, please review our figure guidelines: https://journals.plos.org/plosone/s/figures

You may also use PLOS’s free figure tool, NAAS, to help you prepare publication quality figures: https://journals.plos.org/plosone/s/figures#loc-tools-for-figure-preparation.

NAAS will assess whether your figures meet our technical requirements by comparing each figure against our figure specifications.

# Author comment: Author response to Decision Letter 1

**DOI:** 10.1371/journal.pone.0360298.r003

15 Jul 2026

Response to Review

We thank you and both reviewers for the constructive and detailed feedback. We have substantially revised the manuscript in line with the editorial and reviewer comments.

The principal change is structural. We agree the original framing made the manuscript feel heterogeneous and have reorganized the manuscript into the standard Introduction, Materials and Methods, Results, and Discussion format requested by the Editor and Reviewer #1. In doing so, we have refocused the manuscript explicitly as a two-stage methodology evaluation throughout: (i) laboratory benchmarking of the Wyze v3 against the GardePro A3S (now Fig 1, the central result), and (ii) field validation along three independent methodological axes (hardware survival under tropical conditions, sensitivity-versus-event-capture trade-off, and ecological validity of laboratory-measured behavior; now Fig 2). Behavioral observations not directly relevant to technical validation (sex differences in caregivers, canister preferences, opportunistic rare-behavior captures, Cruziohyla craspedopus observations) have been moved to Supporting Information. This reframing addresses the concern that the original manuscript read as "two completely different papers woven together".

We respond to each point below. Reviewer text is shown in italics; our responses follow each point. Page and line numbers refer to the revised manuscript with tracked changes.

Response to the Editor

"I agree with the reviewers that the current organization of this manuscript is confusing. I believe it would greatly benefit from following a standard intro, materials and methods, results, and discussion format. The current paper also reads like two completely different papers woven together."

Response: We agree on the structural point and have fully reorganized the manuscript into Introduction, Materials and Methods, Results, and Discussion. On the substantive concern about "two papers woven together," we have addressed this by reframing the field work as a methodological validation stage (along hardware, sensitivity, and ecological-validity axes) rather than as a separate behavioral case study. The Introduction now explicitly states that two complementary lines of evidence are required to establish a camera system for ectotherm behavioral research: laboratory benchmarking against an established system, and field validation under real-world conditions. The remainder of the manuscript follows this two-stage logic. The senior author has been closely involved in this revision.

"Figure 1 contains the central results of the study. The materials, methods and results related to this are the only part of the study that needs to be described in detail. Most of the results described in figures 2-4 can be moved to the supplement, since the only point that they support is that the Wyze 3 can be used to capture a wide variety of behavioral data. The details of the behavioral data captured don't really matter in the context of the central question."

Response: We have moved the behavioral-illustration content to the Supporting Information, in line with the Editor's recommendation. Original Fig 3 panels showing sex differences in caregivers and canister preferences are now S1 Fig (parental investment across offspring stage and environments). Original Fig 4 (opportunistic captures of rare behaviors) is now S2 Fig, and original Fig 5 (night recordings of Cruziohyla craspedopus) is now S4 Fig. We have retained two figures from the original manuscript in the main text in revised form, as they are essential to the field validation rather than illustrative of behavioral diversity: revised Fig 2 documents the field installation, the close-range refocus modification (hardware-survival evidence; this was original Fig 2), and the lab-versus-field comparison of male R. variabilis parental visit duration as the ecological-validity test (Fig 2C), simplified to two groups (captive males vs. wild males). Fig 1 (laboratory benchmarking) remains the primary result of the study.

"The materials, methods, and results related to the frog study system need to be much more clearly distinguished from the materials, methods, and results related to testing whether the Wyze 3 shows similar effectiveness to a more expensive system. I would suggest creating a box that describes the frog study-system used for proof-of-concept analyses, to separate this text out from the material related to the central questions."

Response: We have added a dedicated "Anuran study systems" subsection to Materials and Methods, which compactly describes the three frog species used: Dendrobates tinctorius as the focal species for laboratory benchmarking, Ranitomeya variabilis as the focal species for field validation, and Cruziohyla craspedopus for illustrative nocturnal observations in Supporting Information. The Materials and Methods of the main text is now organized around the two-stage evaluation: Camera systems compared, Laboratory benchmarking, Field validation (with the three validation criteria stated explicitly), Behavioral scoring, Statistical analysis, Camera setup, and Anuran study systems. This separates the methods directly required for the camera evaluation from the study-system descriptions, which are contained in the Anuran study systems subsection.

"The results in figure 1 also need to be made much clearer. For example, it is not immediately obvious that most of them differed significantly. I would suggest that you label the sets of observations that are significantly different using standard notation (e.g., an asterisk), with the test statistics and P-values from analyses also included in each section of the figure."

Response: We have revised Fig 1 accordingly. Each panel now displays the relevant significance marker directly above the compared groups (NS for not significant; *** for p < 0.001), with the test statistic and exact p-value printed in the panel (panel D: Wilcoxon rank-sum test, NS; panel I: Wilcoxon signed-rank test with continuity correction, V = 631, p < 0.001). The figure legend lists test details for each panel. Following Reviewer #1, p-values are reported in italics, and values below 0.001 are reported as p < 0.001 rather than in exponential form (the value originally reported as p = 2.54E-05).

"You also need to explain much more directly whether and why these results support the use of the Wyze 3 over the GardePro. It appears that the Wyze 3 often collected data that was significantly different from the GardePro. However, in most of those cases it actually collected more data, with the exception of the observations in Figure 1G. Using a standard format where (Results) you describe the results and then (Discussion) explain to us what conclusions you draw from the results would likely help with this."

Response: We have added a dedicated Discussion paragraph that explicitly addresses this. We state on which axes the Wyze outperforms GardePro (event count in continuous mode; behavior lost per event; behavior recorded per detected event in trigger mode) and explicitly resolve the apparent inconsistency in Fig 1G: although the Wyze produces fewer triggers in trigger mode, the event-matched analysis in Fig 1H to 1I shows that this corresponds to fewer-but-longer recordings, not to missed events. Combined with the roughly nine-fold lower unit cost, we interpret the result as supporting Wyze preference for laboratory and small-scale field studies of small ectotherms. We also state the two axes on which Wyze is not preferred (IP65 vs. IP66 weather rating; USB vs. AA power).

"One small point is that on the title page, the same symbol is used for the corresponding author and shared lead authorship."

Response: We corrected this issue. Shared first authorship is now denoted by the dagger symbol and the corresponding author by an asterisk, with separate footnotes.

Response to Journal Requirements

"Please ensure that your manuscript meets PLOS ONE's style requirements..."

Response: The manuscript has been reformatted to PLOS ONE templates for the title page, main body, and figure legends.

"In your Methods section, please provide additional information regarding the experiments involving animals and ensure you have included details on (1) methods of sacrifice, (2) methods of anesthesia and/or analgesia, and (3) efforts to alleviate suffering."

Response: We have added the following statement to the Camera setup and modifications methods subsection: "No animals were sacrificed or anesthetized in this study; cameras recorded freely behaving animals in their home enclosures and in natural habitats, and no procedures requiring anesthesia or analgesia were performed."

"We note that the grant information you provided in the 'Funding Information' and 'Financial Disclosure' sections do not match."

Response: This has been corrected on the submission portal.

"Please state what role the funders took in the study."

Response: We have added the requested statement: "The funders had no role in study design, data collection and analysis, decision to publish, or preparation of the manuscript."

"We note that Figures 1 and 2 in your submission contain copyrighted images."

Response: In Figure 1, the Wyze copyrighted image was removed in the revision. In Figure 2, we took pictures of the equipment in our lab rather than using copyrighted images. See the original photo below:

"Please include captions for your Supporting Information files at the end of your manuscript, and update any in-text citations to match accordingly."

Response: Supporting Information captions for S1 to S4 Figs and S1 to S6 Videos are now provided at the end of the manuscript. All in-text references have been updated to the new S-numbering.

Response to Reviewer #1

"The introduction is very brief and does not describe the importance of your case study. Why did you choose this species and behavior? What were your predictions and aims for this study?"

Response: We have expanded the Introduction. The final paragraph now states the three specific benchmarking questions we ask (event count, recording duration per event, missed-event rate) and the three field-validation criteria (hardware survival, sensitivity-versus-capture trade-off, ecological validity). Ranitomeya variabilis was selected for field validation because its parental behavior involves transient, hard-to-observe events occurring at small spatial scales, which is exactly the use case home security cameras would need to capture, and because its population at Inselberg, French Guiana, allowed paired laboratory and field deployment under extreme environmental conditions. This rationale is now stated explicitly in the Field validation subsection of Materials and Methods.

"I understand that the PLOS ONE submission guidelines do not state that the materials and methods section needs to come before results and discussion, but these sections would benefit from a restructured organization that is smooth and easy to follow."

Response: We agree. The manuscript has been restructured into separate Introduction, Materials and Methods, Results, and Discussion sections. Results are now strictly limited to what we observed; interpretation, mechanism, and recommendations are deferred to Discussion.

"Everything from the beginning of the results and discussion section to line 122 on page 9 could be described as methods rather than results or discussion."

Response: We agree and have changed this in the revised version. Camera class comparison criteria, benchmarking design (96 h periods, sensitivity settings, focal area), field-validation criteria, behavioral scoring rules, and the rationale for non-parametric tests are now all in Materials and Methods (subsections: Camera systems compared, Laboratory benchmarking, Field validation, Behavioral scoring, Statistical analysis). The Results section opens with the technical comparison (Table 1) and proceeds to the laboratory benchmarking and then the three field-validation results in sequence.

"I specifically focus on the paragraph starting on line 214 as an example of improving the overall structure of this study. In one paragraph, you describe your methods of measurement, report descriptive and analyzed statistics, and then interpret these results claiming that you have a novel finding on R. variabilis. While I appreciate the concise nature of such a paragraph, I lack the context as to why you set your methods this way, why you chose this test, and what the importance of this finding is to the species or goals of investigating this species."

Response: That paragraph has been decomposed across the new structure: (i) the methodological design (paired nursery and adjacent water pool; daily monitoring; lab vs. field comparison) is now in the Field validation subsection of Materials and Methods; (ii) the descriptive statistics and the Kruskal-Wallis rank sum test result are reported in Results under "Field validation 3: ecological validity of laboratory-measured behavior" and displayed in Fig 2C; (iii) the interpretation is reported in Discussion, now reframed as a methodological result (laboratory measurements generalize to natural conditions) rather than a behavioral finding about the species. The original behavioral-finding content (sex differences in caregivers; canister preferences) has been moved to S1 Fig as it is not central to the camera validation.

"For your statistical reports, please clarify why you chose each non parametric test as a best fit for your data."

Response: We have added a Statistical analysis subsection to Materials and Methods explaining the choice: non-parametric tests were used because Shapiro-Wilk tests indicated non-normality of the distributions of behavioral event durations and visit lengths, and because sample sizes were modest. For paired event-duration comparisons between Wyze and GardePro in trigger mode, the Wilcoxon signed-rank test with continuity correction was used because data were paired by event. For the lab-versus-field comparison of male parental visit duration, a Kruskal-Wallis rank sum test was used. The choice of test for each comparison is also restated at the point of use in Results and in the figure legends.

"Additionally, please italicize your p values and I strongly recommend reporting all values below 0.001 as <0.001. On Line 158, you report a p value left in exponential form, which makes it less accessible and clear."

Response: All p values are now italicized throughout the text, figure legends, and tables. The value previously reported as p = 2.54E-05 is now reported as p < 0.001. Test statistics and the abbreviation NS are italicized where used.

Response to Reviewer #2

"One issue I noticed was that a reviewer requested statistical tests for comparisons between two cameras; the authors said that they added tests, but I saw only one test in the whole article."

Response: Every comparison reported in Results is now accompanied by an explicit test, test statistic, p-value, and reference to the Methods subsection in which the test choice is justified. For Fig 1, the comparisons reported are: number of observations in continuous mode (descriptive, Fig 1C); observation length in continuous mode (Wilcoxon rank-sum test, W = 3315.5, NS, Fig 1D); seconds lost per recording in continuous mode (descriptive mean, Fig 1E); proportion of recording time with behavior (descriptive, Fig 1F); number of triggers in trigger mode (descriptive, Fig 1G); event-matched detection concordance (descriptive, Fig 1H); and event-matched behavior duration (Wilcoxon signed-rank test with continuity correction, V = 631, p < 0.001, Fig 1I). For the field validation ecological-validity test (Fig 2C), male parental visit duration was compared between captive and wild R. variabilis using a Kruskal-Wallis rank sum test (χ² = 0.42, p = 0.81, NS).

"The article is interesting and is well written, although the style is heterogeneous. The article is partially a methodology study (of using simple cameras for the study of animal behavior) and partially a field report of frog behavior (Dendrobates tinctorius and Ranitomeya variabilis). For a methodology article, the description of frog behavior could be greatly shortened."

Response: We agree the original framing was heterogeneous. We have restructured the manuscript as a two-stage methodology evaluation throughout: laboratory benchmarking (stage 1) followed by field validation (stage 2). The field validation is now explicitly framed as testing three methodological criteria (hardware survival, sensitivity-versus-capture trade-off, ecological validity of laboratory measurements) rather than as a behavioral study of R. variabilis. The behavioral observations not central to validation (sex differences in caregivers, canister preferences) have been moved to S1 Fig; opportunistic rare-behavior captures are now S2 Fig; and Cruziohyla craspedopus observations are now S4 Fig. The frog species used are now summarized compactly in an "Anuran study systems" subsection of Materials and Methods.

"Table 1 is the heart of the manuscript and provides the main information in the article."

Response: In the revised structure, Table 1 (technical comparison of seven camera systems) and Fig 1 (Wyze v3 vs. GardePro A3S benchmarking) together carry the central results. The Results section opens with Table 1 and proceeds directly to the empirical benchmarking presented in Fig 1, before turning to the field validation.

"On page 4, line 89, the subject of the sentence is plural ('both home security cameras') but the verb is conjugated in the singular ('has')."

Response: Corrected: "both home security cameras Wyze v3 and Tapo have multiple degrees of night vision capabilities..."

"On page 5, line 114, prices are said to range from upwards of $450 USD per camera to $35 USD; although the passage is not grammatically wrong, ranges are usually reported from lower to higher."

Response: Corrected. The sentence now reads "Camera prices ranged from $35 USD to upwards of $450 USD per camera."

"On page 10, line 172, temperatures in the field are reported to range from 20.5°C to 50°C; this may be correct, but 50°C is a very high temperature; was this measured in the air (unlikely) or on the surface of an object under direct sunlight (more likely)?"

Response: 50°C was a surface-temperature measurement. The temperature was recorded at exposed granite-rock surfaces in direct sunlight on the Inselberg of the Nouragues Nature Reserve. We refer to substrate temperature rather than air temperature because this measure is more biologically relevant for these frogs as they are in direct contact with the substrate, making substrate temperature a more accurate representation of the thermal conditions they experience than the temperature of the surrounding air.Even higher surface-temperatures (up to 75°C) were reported for this field site (Sarthou et al. 2009; ref 34). The text in both the Introduction (Field validation paragraph) and the Methods now clarifies this and we added a Supplementary Figure (Fig. S3) depicting the measurement location as well as the temperature profile recorded by the logger.

Attachment Submitted filename: Response_to_Reviewers_v5.docx

# Peer review: Decision Letter 1

**DOI:** 10.1371/journal.pone.0360298.r004

**Authors:** Patrick Stephens

12 Aug 2026

Dear Dr. O'Connell,

Please submit your revised manuscript by Sep 26 2026 11:59PM. If you will need more time than this to complete your revisions, please reply to this message or contact the journal office at plosone@plos.org. When you're ready to submit your revision, log on to https://www.editorialmanager.com/pone/ and select the 'Submissions Needing Revision' folder to locate your manuscript file.

- A letter that responds to each point raised by the academic editor and reviewer(s). You should upload this letter as a separate file labeled 'Response to Reviewers'.
- A marked-up copy of your manuscript that highlights changes made to the original version. You should upload this as a separate file labeled 'Revised Manuscript with Track Changes'.
- An unmarked version of your revised paper without tracked changes. You should upload this as a separate file labeled 'Manuscript'.

If you would like to make changes to your financial disclosure, please include your updated statement in your cover letter. Guidelines for resubmitting your figure files are available below the reviewer comments at the end of this letter.

If applicable, we recommend that you deposit your laboratory protocols in protocols.io to enhance the reproducibility of your results. Protocols.io assigns your protocol its own identifier (DOI) so that it can be cited independently in the future. For instructions see: https://journals.plos.org/plosone/s/submission-guidelines#loc-laboratory-protocols. Additionally, PLOS ONE offers an option for publishing peer-reviewed Lab Protocol articles, which describe protocols hosted on protocols.io. Read more information on sharing protocols at https://plos.org/protocols?utm_medium=editorial-email&utm_source=authorletters&utm_campaign=protocols.

As the corresponding author, your ORCID iD is verified in the submission system and will appear in the published article. PLOS supports the use of ORCID, and we encourage all coauthors to register for an ORCID iD and use it as well. Please encourage your coauthors to verify their ORCID iD within the submission system before final acceptance, as unverified ORCID iDs will not appear in the published article. *Only*  the individual author can complete the verification step; PLOS staff *cannot*  verify ORCID iDs on behalf of authors.

We look forward to receiving your revised manuscript.

Kind regards,

Patrick R Stephens, Ph.D.

Academic Editor

PLOS One

Journal Requirements:

If the reviewer comments include a recommendation to cite specific previously published works, please review and evaluate these publications to determine whether they are relevant and should be cited. There is no requirement to cite these works unless the editor has indicated otherwise.

Please review your reference list to ensure that it is complete and correct. If you have cited papers that have been retracted, please include the rationale for doing so in the manuscript text, or remove these references and replace them with relevant current references. Any changes to the reference list should be mentioned in the rebuttal letter that accompanies your revised manuscript. If you need to cite a retracted article, indicate the article’s retracted status in the References list and also include a citation and full reference for the retraction notice.

Additional Editor Comments:

This manuscript has been vastly improved compared to the original submission.  Unfortunately, the original reviewer that made the most suggestions on the original submission became unavailable to assess the revision.  However, we have secured another well qualified reviewer, that has a number of additional suggestions for clarity.  Since no new analyses will be required, it should be a relatively straightforward revision.

[Note: HTML markup is below. Please do not edit.]

Reviewers' comments:

Reviewer's Responses to Questions

**Comments to the Author**

Reviewer #2: All comments have been addressed

Reviewer #3: (No Response)

**********

2. Is the manuscript technically sound, and do the data support the conclusions?

Reviewer #2: Yes

Reviewer #3: Yes

**********

3. Has the statistical analysis been performed appropriately and rigorously?

Reviewer #2: Yes

Reviewer #3: No

**********

4. Have the authors made all data underlying the findings in their manuscript fully available?

The PLOS Data policy

Reviewer #2: Yes

Reviewer #3: Yes

**********

5. Is the manuscript presented in an intelligible fashion and written in standard English?

Reviewer #2: Yes

Reviewer #3: No

**********

Reviewer #2: (No Response)

Reviewer #3: I think overall this is an excellent study and does valuable work advocating for understudied species and science accessibility. My issues with the manuscript are primarily in terms of organization. While there are marked improvements from the previous version, there are issues throughout where information appropriate for the introduction or methods section is not disclosed until the results or discussion.

I think the introduction would benefit from more detail to provide readers with necessary context. Some statements require citations (e.g. line 40 that Global South has much of ectotherm biodiversity). The paragraphs describing the differences between camera traps and home security cameras (lines 46-60) are lacking some potentially relevant details, such as the difference between how camera systems generally record daytime and nighttime videos. If there is some form of IR that can detect ectotherms, this should be mentioned in the introduction. While it makes sense that the introduction would focus on the differences between the two camera sources, I think the focal species and behaviors of interest should be described in the introduction, briefly, and in the context of what observational challenges would be directly improved by the results of this study. This information is present in Field validation subsection of methods, currently. The author's introduction should end with their hypotheses about how the two camera systems will perform in the lab and wild.

The methods section has discrete subheaders, which is great and a marked improvement in organization from the previous version. However, I think some of them would benefit from being expanded and reorganized. The authors might consider having a multistudy organization such that the camera systems comparison and laboratory study is reported in full (methods, results, discussion) before reporting on the wild study (with another methods, results, and discussion section), where the emphasis can remain on how the camera systems differed in terms of accurate event recording. The observations of C. craspedopus, unless they reveal something critical about Wayze' nighttime capacities not captured in the other two experiments, may be better off saved for a future manuscript. If this reorganization suggestion is pursued, anuran study systems can be separated and organized with the corresponding experiment. Otherwise, I think it would provide better narrative flow to place that section earlier in the methods, before behavioral coding. Environmental factors (brightness, humidity, etc) seem relevant in this case and could be included when describing the terrarium. Behavioral scoring would benefit from being accompanied by an ethogram of the behaviors studied. The amount of footage and total number of events (and total count per behavior) recorded by each camera system should be included. Finally, the "Statistical analysis" section should be the final section of methods and precede the results.

Given that no statistical analysis is necessary to describe the differences between the camera systems, Table 1 and lines 193-203 would fit better in the beginning of the methods section, under the "Camera systems compared" subheader (line 76), where the content of that table is first discussed. In the paragraph of lines 211-220, the authors argue that "Wyze v3 detects more behavioral events than GardePro A3S in continuous mode" however I don't see a p value to support this statement. If the loss of 21 seconds per recording led to statistically significant differences in numbers of events recorded, the test determining that should be reported. Along that line of thought, figure 1C and 1D would benefit from denoting significance level between camera types. Lines 228-237 are a lovely summary of the preceding results and are better suited as the first paragraph of the discussion.

Lines 257-306 return to the heterogenous organization and are a mixture of information appropriate for methods, results, and discussion. The authors should try to keep descriptions of field sites and procedure in the methods, limit the results to the outcomes of statistical analysis (or demographics of the dataset), and save the majority of interpretations or any mentions of alternative solutions to limitations for the discussion. The discussion in lines 388-404 is excellent and demonstrates the importance of this work. Information about camera systems differences and similarities might benefit from being described in the context of the specific frog behaviors observed by the study.

Typographical or grammatical errors:

line 35: "bth" rather than "both"

line 78: "...the suitability" it is more appropriate to use "their suitability" in this case

lines 108-113: This is a complex, multi-clause sentence. Consider breaking up into two or three sentences. Also, "air-to-surface temperature" is a bit unclear; is this the range of both air and surface temperatures? Consider reporting each individually.

line 195: The colon can be replaced by a period. As it stands, 194-197 is another run on sentence.

line 216-217: Consider breaking this up into two sentences (replaced "but" with "however")

line 286: difficult-to-observe; I think the hyphens are unnecessary

line 333: unnecessary comma

line 347: "identifying" should be "identify"

line 372: Again, consider replacing colon with period and making two separate sentences

**********

what does this mean?). If published, this will include your full peer review and any attached files.

If you choose “no”, your identity will remain anonymous but your review may still be made public.

**Do you want your identity to be public for this peer review?**  For information about this choice, including consent withdrawal, please see our Privacy Policy

Reviewer #2: No

Reviewer #3: No

**********

[NOTE: If reviewer comments were submitted as an attachment file, they will be attached to this email and accessible via the submission site. Please log into your account, locate the manuscript record, and check for the action link "View Attachments". If this link does not appear, there are no attachment files.]

To ensure your figures meet our technical requirements, please review our figure guidelines: https://journals.plos.org/plosone/s/figures

You may also use PLOS’s free figure tool, NAAS, to help you prepare publication quality figures: https://journals.plos.org/plosone/s/figures#loc-tools-for-figure-preparation.

NAAS will assess whether your figures meet our technical requirements by comparing each figure against our figure specifications.

# Author comment: Author response to Decision Letter 2

**DOI:** 10.1371/journal.pone.0360298.r005

31 Aug 2026

Response to the reviewers

We thank the reviewers for their thoughtful and constructive comments, which have helped us to improve the manuscript. Below, we detail the changes made in response to each comment. Below, the reviewers’ comments are shown in black, followed by our responses in blue. For the reviewer’s convenience, all lines refer to the untracked version of the manuscript. We also found a small figure 1 error, which is now corrected (Fig 1H was accidentally labeled Fig1I, and Fig 1I was accidentally labeled Fig1J).

Reviewer #3:

I think overall this is an excellent study and does valuable work advocating for understudied species and science accessibility. My issues with the manuscript are primarily in terms of organization. While there are marked improvements from the previous version, there are issues throughout where information appropriate for the introduction or methods section is not disclosed until the results or discussion.

In the revised version, we have addressed this comment through the reorganization described point-by-point below.

I think the introduction would benefit from more detail to provide readers with necessary context. Some statements require citations (e.g. line 40 that Global South has much of ectotherm biodiversity).

We thank the reviewer for this feedback. For clarity, we have added detail and citations, specifically the following:

3. Martin PC, Bateson P. Measuring behaviour: An introductory guide. Cambridge, England: Cambridge University Press; 1986.

11. Welbourne DJ, Claridge AW, Paull DJ, Lambert A. How do passive infrared triggered camera traps operate and why does it matter? Breaking down common misconceptions. Remote Sens Ecol Conserv. 2016;2: 77–83.

12. Welbourne DJ, Paull DJ, Claridge AW, Ford F. A frontier in the use of camera traps: surveying terrestrial squamate assemblages. Remote Sens Ecol Conserv. 2017;3: 133–145.

13. Rovero F, Zimmermann F, Berzi D, Meek P. “Which camera trap type and how many do I need?” A review of camera features and study designs for a range of wildlife research applications. Hystrix-italian Journal of Mammalogy. 2013;24: 148–156.

14. Collen B, Ram M, Zamin T, McRae L. The tropical biodiversity data gap: Addressing disparity in global monitoring. Trop Conserv Sci. 2008;1: 75–88.

15. Mugerwa B, Niedballa J, Planillo A, Sheil D, Kramer-Schadt S, Wilting A. Global disparity of camera trap research allocation and defaunation risk of terrestrial mammals. Remote Sens Ecol Conserv. 2024;10: 121–136.

16. Whytock RC, Suijten T, van Deursen T, Świeżewski J, Mermiaghe H, Madamba N, et al. Real-time alerts from AI-enabled camera traps using the Iridium satellite network: a case-study in Gabon, Central Africa. Ecology. bioRxiv; 2021. Available: https://www.biorxiv.org/content/10.1101/2021.11.10.468078v1.full.pdf

17. Radke RJ, Andra S, Al-Kofahi O, Roysam B. Image change detection algorithms: a systematic survey. IEEE Trans Image Process. 2005;14: 294–307.

37. Meek PD, Ballard G-A, Fleming PJS, Schaefer M, Williams W, Falzon G. Camera traps can be heard and seen by animals. PLoS One. 2014;9: e110832.

The paragraphs describing the differences between camera traps and home security cameras (lines 46-60) are lacking some potentially relevant details, such as the difference between how camera systems generally record daytime and nighttime videos.

This has been expanded upon and clarified in lines 39-44 and 54-59.

If there is some form of IR that can detect ectotherms, this should be mentioned in the introduction.

There have been successful PIR recordings in mostly larger squamates such as crocodilians. In smaller squamates, PIR recording has been successfully done with a modified version of PIR called camera overhead augmented temperature (COAT) in Welbourne et al. 2013 (citation 12 above reviews this) which leverages the difference of squamate surface heat (similar to environment) compared to a cork background. Lines 41-43 hope to clarify this point.

While it makes sense that the introduction would focus on the differences between the two camera sources, I think the focal species and behaviors of interest should be described in the introduction, briefly, and in the context of what observational challenges would be directly improved by the results of this study. This information is present in Field validation subsection of methods, currently.

In response to this concern, a new paragraph was added to the end of the Introduction describing both study species (D. tinctorius and R. variabilis) and explaining their respective advantages for addressing the research question.

The author’s introduction should end with their hypotheses about how the two camera systems will perform in the lab and wild.

A new closing paragraph was added to the introduction stating the hypotheses of this work.

The methods section has discrete subheaders, which is great and a marked improvement in organization from the previous version. However, I think some of them would benefit from being expanded and reorganized.

We addressed this point via the restructuring described below.

The authors might consider having a multistudy organization such that the camera systems comparison and laboratory study is reported in full (methods, results, discussion) before reporting on the wild study (with another methods, results, and discussion section), where the emphasis can remain on how the camera systems differed in terms of accurate event recording.

We thank the reviewer for this helpful suggestion. Following this recommendation, we reorganized the manuscript using a multi-study structure. The manuscript is now divided into Study 1: Camera Comparison and Laboratory Benchmarking and Study 2: Field Validation, each with its own Results and Discussion sections. We retained a shared Materials and Methods section because both studies rely on common approaches for behavioral coding and statistical analyses. However, this section has been subdivided into methods common to both studies, methods and study system specific to Study 1, and methods and study system specific to Study 2.

The observations of C. craspedopus, unless they reveal something critical about Wayze’ nighttime capacities not captured in the other two experiments, may be better off saved for a future manuscript.

The C. craspedopus study system and all associated body text have been removed.

If this reorganization suggestion is pursued, anuran study systems can be separated and organized with the corresponding experiment. Otherwise, I think it would provide better narrative flow to place that section earlier in the methods, before behavioral coding.

We followed the reviewer’s advice and introduced D. tinctorius under Study 1 ("Anuran study system in the laboratory") and R. variabilis under Study 2 ("Anuran study system in the field").

Environmental factors (brightness, humidity, etc) seem relevant in this case and could be included when describing the terrarium.

We followed the reviewer’s advice, and this has been added under Study 1 (“Anuran study system in the laboratory”).

Behavioral scoring would benefit from being accompanied by an ethogram of the behaviors studied.

For simplicity, frog attendance was measured as when the frog was present or absent for camera comparisons. In tadpole recordings, attendance was measured as the frog having the majority of its body (snout to vent) within the bromeliad or canister nursery measurements. For more qualitative behaviors not mentioned in the main text, we recorded the four qualitative and highly stereotyped behaviors described previously in Brown et al., 2008: egg care, tadpole transport, mating, pool visits (no tadpole), and pool visits (tadpole visits). Mating is when a calling male and female enter the canister together, huddling. Confirmation of mating is visually seen with the presence of an egg laid by the female. Egg care is the subsequent behavior of both sexes visiting the egg, often paired with huddling within a body’s length or on top of the egg itself. This is assumed to be parents hydrating via urination and/or guarding eggs. Tadpole transport is when a tadpole wriggles onto the adult’s back, which is visually confirmed with its body on the adult’s back. Tadpole transport concludes when the tadpole is physically off of the adult’s back. These observations are encoded in the Supplementary Data spreadsheet ‘CombinedVisits’ in the column ‘behaviors’.

The amount of footage and total number of events (and total count per behavior) recorded by each camera system should be included.

We described this in passing in ‘Laboratory benchmarking: Wyze v3 detects more behavioral events than GardePro A3S in continuous mode’, but we agree with reviewers that this section would benefit from explicit descriptions of the footage and events recorded and therefore has been added.

Finally, the "Statistical analysis" section should be the final section of methods and precede the results.

Statistical analysis is now the last general Methods subsection (under "Studies 1 & 2"), immediately before the Results.

Given that no statistical analysis is necessary to describe the differences between the camera systems, Table 1 and lines 193-203 would fit better in the beginning of the methods section, under the "Camera systems compared" subheader (line 76), where the content of that table is first discussed.

We did not address this comment, as we respectfully disagree with the suggested change. Descriptive results are commonly reported without statistical analysis, for example in case studies, systematic reviews, or methodological and technical comparisons. The table summarizes the outcome of our systematic comparison of the camera systems and therefore represents a result of Study 1 rather than a description of the methods used. The comparison also informed the choice of camera model and will be useful to researchers selecting a camera system that best matches their study question. Accordingly, this comparison was considered one of the most important components of the study by other reviewers. We therefore consider Table 1 and the corresponding text better suited to the Results section.

In the paragraph of lines 211-220, the authors argue that "Wyze v3 detects more behavioral events than GardePro A3S in continuous mode" however I don’t see a p value to support this statement. If the loss of 21 seconds per recording led to statistically significant differences in numbers of events recorded, the test determining that should be reported.

This has been clarified to soften the language of this claim, given the count difference was statistically insignificant. However, it is difficult to run a statistical analysis for recording loss with Wyze in continuous mode vs GardePro, as our a priori expectations of data loss cannot be 50/50 with one camera recording 24/7 with 0 seconds of data loss. Thus, we added a comparison of the proportion of data lost (60/68 of events detected by GardePro experienced data loss; 88%, 95% CI 78-95%).

Along that line of thought, figure 1C and 1D would benefit from denoting significance level between camera types.

We thank the reviewer for this suggestion and have added “NS” to both panels to specify non significance.

Lines 228-237 are a lovely summary of the preceding results and are better suited as the first paragraph of the discussion.

This summary paragraph was merged with a sentence about the previous use of security cameras and now opens the Study 1 Discussion.

Lines 257-306 return to the heterogenous organization and are a mixture of information appropriate for methods, results, and discussion. The authors should try to keep descriptions of field sites and procedures in the methods, limit the results to the outcomes of statistical analysis (or demographics of the dataset), and save the majority of interpretations or any mentions of alternative solutions to limitations for the discussion.

We addressed the reviewers' concern by restructuring the manuscript. Specifically, field-site and procedural descriptions were moved into the Methods for Study 2, Results now report outcomes only. The interpretation of the results has been moved into the Study 2 Discussion, alongside the limitations.

The discussion in lines 388-404 is excellent and demonstrates the importance of this work.

We thank the reviewer and moved this general part of the discussion to a new “Conclusions” part with preserved verbatim.

Information about camera systems differences might benefit from being described in the context of the specific frog behaviors observed by the study.

We added an introductory paragraph to the discussion of the field study. In the revised version, it now reads:

“Differences among camera systems in both event detection and recording duration can affect which ectotherm behaviors are successfully captured and documented. Tadpole nurseries of small poison frogs, in this case bromeliad leaves, cannot support the weight of a trail camera such as the GardePro, which would therefore require additional heavy mounting equipment, such as tripods, to be carried into the field. The Wyze v3's lower missed-event rate and longer recorded duration per detected event, as established in the laboratory benchmarking, are directly relevant to monitoring poison frog behavior in the field. Many nursery visits are short in duration and could be missed entirely by a camera with a shorter trigger-to-recording window. Conversely, longer recording durations may improve the detection and documentation of more prolonged behaviors that could otherwise be truncated, such as heterospecific intrusions or the previously undescribed offspring-wiping event.”

Typographical or grammatical errors:

line 35: "bth" rather than "both"

line 78: "...the suitability" it is more appropriate to use "their suitability" in this case

lines 108-113: This is a complex, multi-clause sentence. Consider breaking up into two or three sentences. Also, "air-to-surface temperature" is a bit unclear; is this the range of both air and surface temperatures? Consider reporting each individually.

line 195: The colon can be replaced by a period. As it stands, 194-197 is another run on sentence.

line 216-217: Consider breaking this up into two sentences (replaced "but" with "however")

line 286: difficult-to-observe; I think the hyphens are unnecessary

line 333: unnecessary comma

line 347: "identifying" should be "identify"

line 372: Again, consider replacing colon with period and making two separate sentences

All typographical errors mentioned by reviewer 3 were corrected as suggested.

Reviewer #2: answered “Yes” to all assessment questions and provided no specific comments requiring a response.

Attachment Submitted filename: Response to reviewer v6.docx

# Peer review: Decision Letter 2

**DOI:** 10.1371/journal.pone.0360298.r006

**Authors:** Patrick Stephens

24 Sep 2026

Home security cameras as a tool for behavior observations and science affordability

PONE-D-26-12504R2

Dear Dr. O'Connell,

We’re pleased to inform you that your manuscript has been judged scientifically suitable for publication and will be formally accepted for publication once it meets all outstanding technical requirements.

Within one week, you’ll receive an e-mail detailing the required amendments. When these have been addressed, you’ll receive a formal acceptance letter and your manuscript will be scheduled for publication.

An invoice will be generated when your article is formally accepted. Please note, if your institution has a publishing partnership with PLOS and your article meets the relevant criteria, all or part of your publication costs will be covered. Please make sure your user information is up-to-date by logging into Editorial Manager at Editorial Manager® and clicking the ‘Update My Information' link at the top of the page. For questions related to billing, please contact billing support.

If your institution or institutions have a press office, please notify them about your upcoming paper to help maximize its impact. If they’ll be preparing press materials, please inform our press team as soon as possible -- no later than 48 hours after receiving the formal acceptance. Your manuscript will remain under strict press embargo until 2 pm Eastern Time on the date of publication. For more information, please contact onepress@plos.org.

Kind regards,

Patrick R Stephens, Ph.D.

Academic Editor

PLOS One

Additional Editor Comments (optional):

Reviewers' comments:

Reviewer's Responses to Questions

**Comments to the Author**

Reviewer #3: All comments have been addressed

**********

2. Is the manuscript technically sound, and do the data support the conclusions?

Reviewer #3: Yes

**********

3. Has the statistical analysis been performed appropriately and rigorously?

Reviewer #3: Yes

**********

4. Have the authors made all data underlying the findings in their manuscript fully available?

The PLOS Data policy

Reviewer #3: Yes

**********

5. Is the manuscript presented in an intelligible fashion and written in standard English?

Reviewer #3: Yes

**********

Reviewer #3: This looks great! I am pleased that the multistudy organization I suggested seems to have been a good choice. The authors' explanation for why Table 1 should remain in the result section makes sense, and I support the decision.

**********

what does this mean?). If published, this will include your full peer review and any attached files.

If you choose “no”, your identity will remain anonymous but your review may still be made public.

**Do you want your identity to be public for this peer review?**  For information about this choice, including consent withdrawal, please see our Privacy Policy

Reviewer #3: No

**********

# Editor report: Acceptance letter

**DOI:** 10.1371/journal.pone.0360298.r007

**Authors:** Patrick Stephens

PONE-D-26-12504R2

PLOS One

Dear Dr. O'Connell,

I'm pleased to inform you that your manuscript has been deemed suitable for publication in PLOS One. Congratulations! Your manuscript is now being handed over to our production team.

At this stage, our production department will prepare your paper for publication. This includes ensuring the following:

* All references, tables, and figures are properly cited

* All relevant supporting information is included in the manuscript submission,

* There are no issues that prevent the paper from being properly typeset

You will receive further instructions from the production team, including instructions on how to review your proof when it is ready. Please keep in mind that we are working through a large volume of accepted articles, so please give us a few days to review your paper and let you know the next and final steps.

Lastly, if your institution or institutions have a press office, please let them know about your upcoming paper now to help maximize its impact. If they'll be preparing press materials, please inform our press team within the next 48 hours. Your manuscript will remain under strict press embargo until 2 pm Eastern Time on the date of publication. For more information, please contact onepress@plos.org.

You will receive an invoice from PLOS for your publication fee after your manuscript has reached the completed accept phase. If you receive an email requesting payment before acceptance or for any other service, this may be a phishing scheme. Learn how to identify phishing emails and protect your accounts at https://explore.plos.org/phishing.

If we can help with anything else, please email us at customercare@plos.org.

Thank you for submitting your work to PLOS One and supporting open access.

Kind regards,

PLOS One Editorial Office Staff

on behalf of

Dr. Patrick R Stephens

Academic Editor

PLOS One
